# Ethical issues raised by artificial intelligence and big data in population health: a scoping review

**DOI:** 10.3389/fsoc.2025.1536389

**Published:** 2025-09-09

**Authors:** Vincent Couture, Marie-Christine Roy, Emma Dez, Fannie Tremblay, Jean-Christophe Bélisle-Pipon

**Affiliations:** ^1^Faculty of Nursing, Université Laval, Québec, QC, Canada; ^2^Faculty of Arts and Sciences, Université de Montréal, Montreal, QC, Canada; ^3^Sciences Po, Paris, France; ^4^Faculty of Medicine, Université Laval, Québec, QC, Canada; ^5^Faculty of Health Sciences, Simon Fraser University, Burnaby, BC, Canada

**Keywords:** artificial intelligence, big data, ethics, population health, public health

## Abstract

**Introduction:**

Artificial intelligence systems (AIS) powered by big data (BD) are more and more common in the healthcare sector and many anticipate that they will have a substantial effect on population health. Facing the disruptive potential of these transformations, there is a need to keep the pace with the ethical reflection accompanying the uses of AIS and the BD systems enabling such innovations.

**Methods:**

To carry out this task, we conducted a scoping review of the ethical issues of AIS and BD, in population health, based on 243 scholarly articles.

**Results:**

Our results show the explosion of publications on the subject in recent years. Our qualitative analysis of this literature highlights the potential issues of AIS and BD on the three components of population health: (1) the health outcomes and their distribution in the population and between populations; (2) the patterns of health determinants; (3) the policies and interventions developed to connect the previous components.

**Discussion:**

Our conclusions show the uncertainty of the positive outcomes of these technologies and their potential for unequal distribution. Authors consider that AIS and BD will affect determinants of health either in their understanding and by transforming the structure of these determinants. At last, this review points that the policies and interventions developed to attain population health goals will have to answer to numerous ethical expectations. This review offers a comprehensive mapping of ethical issues raised by the uses of AIS in the global field of population health.

## 1 Introduction

Artificial intelligence systems (AIS) and big data (BD) are of special interest for population health (Mooney and Pejaver, 2018; World Health Organization, 2021). First, they promise an unprecedented capacity to treat and analyze large sets of data coming from vast social assemblages such as populations (Bellazzi, 2014). Second, they generate the possibility for developing large scale health interventions targeting populations or social groups because of their capacity for automation and their potential autonomy from limited human workforce (OECD, 2019; UNESCO, 2024; Dolley, 2018). Beside these promises, it is not clear on which ethical landscape these systems will be deployed (Floridi et al., 2018). To clarify this situation, our aim was to synthetize the state of the ethical reflection on the main ethical challenges raised by the introduction of systems at the intersection of artificial intelligence (AI) and BD from the perspective of population health.

For this task, we apply the definition of population health suggested by (Kindig and Stoddart, 2003). There is no consensus on what “population health” is, but Kindig and Stoddart’s definition offers an accepted base offering the common features implied by this extension of public health. According to these authors, “population health” can be defined as the “the health outcomes of a group of individuals, including the distribution of such outcomes within the group” (Kindig and Stoddart, 2003). It encompasses three interacting components. The first refers to health outcomes and their distribution. The second considers the patterns of health determinants (e.g., healthcare, social environment, physical environment). The third is the interventions and policies connecting the previous components.

In complement, we used the largest definitions of BD and AI to make sure no relevant article was excluded with regards to our research question. That said, both the definitions of BD and AI are porous and somewhat debated. To categorize the particularity of BD, many authors refer to the “three *Vs*” definition: volume, variety and velocity (Vogel et al., 2019; Stylianou and Talias, 2017; Tanti, 2015; Thorpe and Gray, 2015a; Dolley, 2018). A fourth and fifth V are sometimes added for “veracity” (Andanda, 2019; Bellazzi, 2014; Cahan et al., 2019; Liyanage et al., 2014) and “value” (Docherty and Lone, 2015; Lajonchere, 2018; Colloc, 2015; Salas-Vega et al., 2015). Sources of BD for population health include medical (Lee and Yoon, 2017; Wyllie and Davies, 2015; Cheung et al., 2019; Wang and Alexander, 2020) and medical-health data collected in various ways and by multiple devices (Vogel et al., 2019; Andanda, 2019; Mooney and Pejaver, 2018; Leyens et al., 2017; Benke and Benke, 2018; Alemayehu and Berger, 2016; Timmins et al., 2018; Barreto and Rodrigues, 2018; Kern et al., 2016), e.g., electronic health records (EHR) (Gossec et al., 2020), social media (Gossec et al., 2020; Aiello et al., 2020), wearable devices (Gossec et al., 2020), the internet of things (Fornasier, 2019), among others. Data can be personal or proprietary, controlled by the government or available in open data commons (Heitmueller et al., 2014).

BD is used to train and feed AIS. A very general definition of AI designates technologies that can execute tasks by imitating human intelligence (Gossec et al., 2020; Tang et al., 2018; Kerr et al., 2018; Xie et al., 2020). AI includes various approaches such as machine learning (supervised or unsupervised), deep learning, and neural networks (Mooney and Pejaver, 2018; Tang et al., 2018; Xie et al., 2020; Noorbakhsh-Sabet et al., 2019; Lajonchere, 2018; Galetsi et al., 2019; Mohr et al., 2017; Wang and Alexander, 2020; Lanier et al., 2020; Sparrow and Hatherley, 2019). It can take many forms, including some visible on computer screens and others as complex as robots (Fulmer, 2019; Kernaghan, 2014). Together, BD and AI are used in multiple ways to study or improve population health, e.g., health decision-making (Hunt et al., 2020; Conrad et al., 2020; Brill et al., 2019), surveillance (Mbunge, 2020; Budd et al., 2020; Larkin and Hystad, 2017), data analysis and research (Sparrow and Hatherley, 2019; Sanchez M and Sarria-Santamera, 2019; Ladner and Ben Abdelaziz, 2018), and assistive technologies (Kernaghan, 2014; Bennett, 2019; de Graaf et al., 2015; Grigorovich and Kontos, 2020; Miller, 2020; Vollmer Dahlke and Ory, 2020; Althobaiti, 2021; Jiang and Cheng, 2021).

In the next sections, we will defend that the use of AIS fueled by BD may affect paradoxically the three components of population health. It is still uncertain if the benefits of these AIS will balance the numerous risks that these technologies pose for the main goal of population health. We can still doubt whether these expectations will match reality. Hence, our knowledge synthesis offers a roadmap for future ethical assessment of AIS in population health.

## 2 Materials and methods

To achieve our aim, we followed the five stages of the scoping review methodology (Arksey and O’Malley, 2005; Levac et al., 2010; Tricco et al., 2018), starting with the identification of the research question which is: “what are the ethical issues of AIS using BD in population health?”

This question guided us for the next stage which was the identification of relevant studies. With the help of a librarian specialized in reviewing health research evidence, we developed the following research strategy. We conceived a search equation including terms related to the three concepts of our research question: (1) “ethical, legal, and social issues (ELSI),” (2) “population health,” and (3) “AIS and BD technologies” (see Table 1). We selected two databases because of their integration of articles in health sciences and bioethics (Medline) as well as social science and multidisciplinary research (Web of Science). Articles in English and French were included. No restrictions were used for publication date because of the novelty of the topic.

**Table 1:** Search equation.

Concepts
Concept 1. Ethic* OR Bioethic* OR Moral* OR Legal OR Law OR Social OR Politic* OR ELSI OR Governance OR Regulation OR. Empower* OR Inclusive* OR “AI for good” OR Trust OR Privacy OR Accountab* OR Transparen* OR Explainab* OR Fair* OR Discriminat* OR Responsib* OR Integrity OR “Human right*.” “Human right*.” AND Concept 2. “Population health” OR “Populations health” OR “Population’s health” OR “Health of populations” OR “Public health” OR Epidemiology OR “Community health” OR “Health promotion*” OR “Population Polic*” OR “Public Polic*” OR “Health Polic*.” AND Concept 3. “Artificial intelligence” OR “Big Data” OR Algorithm* OR Robot* OR “Machine learning” OR “Representation learning” OR “Deep learning” OR “Supervised learning” OR “Unsupervised learning” OR “Natural language processing” OR Chatbot* OR “Facial recognition” OR “Mobile device*” OR “Internet of things.”

**Figure 1:**
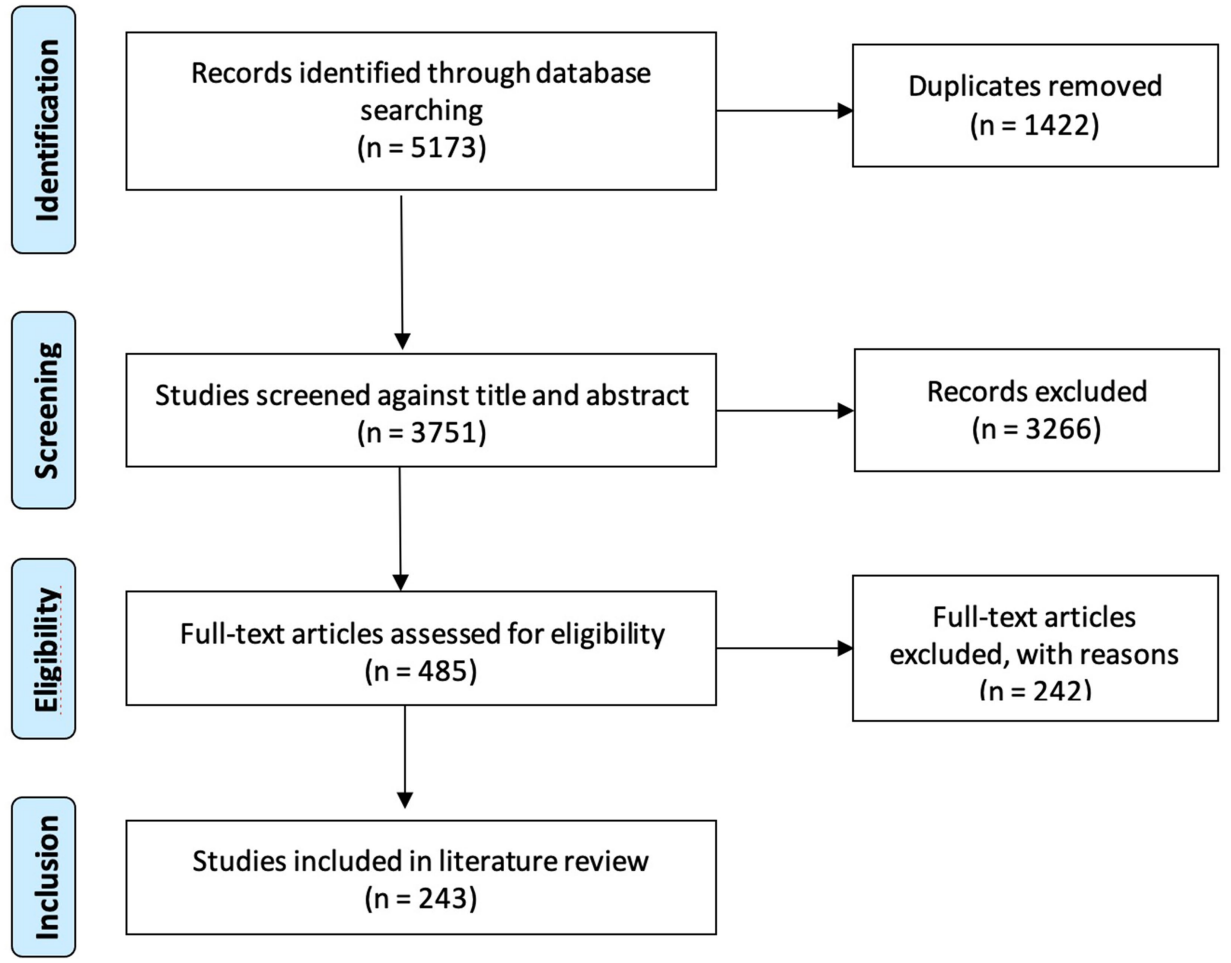
PRISMA flowchart.

Once the strategy was determined, we started the study inclusion stage. For this purpose, we developed selection criteria (see Table 2) to optimize the search and followed the selection process suggested by the PRISMA flowchart (see Figure 1). The first search was conducted June 20, 2020, and it was updated November 24, 2021. The combined searches led to the identification of 5,173 records by screening their title and abstract. Each step of the screening was done by two reviewers (either MCR and JCBP or VC) for each record. After removing duplicates and analyzing the full text, we obtained a final sampling of 243 articles.

**Table 2:** Inclusion and exclusion criteria.

Inclusion criteria	Exclusion criteria
Relates to AI, Big data, Health, and ELSI Language = English or French Document type = peer-reviewed article, commentary, editorial, review, discussion paper, etc.	No mention of AI, Big data, Health or ELSI Language other than English or French Document type = book, book chapter, conference proceedings, reports

For the fourth stage of the review, we charted the data to have a global picture of the literature. Specifically, we look at the year of publication, the region where the first author is located, the academic domain of the article, and the type of technological application described in the article (see Supplementary material).

For the last stage, the articles were qualitatively analyzed following thematic analysis (Braun and Clarke, 2012). With the help of NVivo 12 (QSR International, 2017), we used inductive and deductive coding. Prior to coding, the principles for governing AI mapped in Fjeld et al. (2019) were used as an initial matrix. The codebook was updated as the coding was carried on. To assess intercoder reliability and to produce a first codebook, a subset (5%) of the articles retrieved in the first search were coded by three researchers (VC, JCBP, MCR). Codes were grouped into themes that we discussed within the definition of “population health” suggested by Kindig and Stoddart (2003).

## 3 Results

According to the literature, AIS using BD will generate ethical issues affecting each of the three components of population health: (1) health outcomes and their distribution, (2) the patterns of health determinants, (3) as well as the interventions and policies working on health determinants to create positive outcomes. Table 3 summarize these results.

**Table 3:** Summary of thematic analysis.

Macro themes	Mezzo themes	Micro themes	Examples of issues
Health outcomes and distribution	Uncertain outcomes	Positive health outcomes	Could AIS contribute to optimize healthcare in order to treat a larger quantity of patients?
		Negative health outcomes	Will AI and BD lead to a reductionist understanding of illness?
	Fair distribution of the outcomes	Increase of Health disparities	Will the benefits and burdens of AI and BD technologies be distributed fairly in the population?
		Discrimination and stigmatization	Will these technologies contribute to discriminate communities based on their health status and lead to stigmatization?
		Digital colonialism	Will LMIC received a fair part of the benefits generated by these technologies?
		Digital divide	Will health data and technologies be accessible to all communities?
		Biases in Datasets and Algorithms	What will be the social consequences of the outcomes of biased datasets and algorithm?
Health determinants	Promotion of healthy behaviors	Empowerment and disempowerment	Will these technologies be useful to empower populations and promote positive health behaviors?
		Digital and ethical literacy	Will digital and ethical literacy be taken into account by policymakers?
	Efficient healthcare functioning		What will be the outcomes of the introduction of these technologies on the working conditions of HCP?
	Data control	Data ownership	Who should own health data?
		Data management	How should we arbitrate conflicts between the parties using health data?
		Data accessibility and sharing	Do individuals have a duty to share personal health data for the greater good?
Interventions and policies	Privacy protection	Privacy breaches	What are the risks of reidentification of individuals associated with the use of these technologies?
		Operationalization of privacy standards	Does privacy protection is still relevant in the age of social networks?
	Consent		How to operationalize consent mechanism for population interventions using AI and BD?
	Responsibility, accountability and liability		How to apply the notion of professional responsibility with AIS?
	Transparency		Is there a duty to make AIS transparent?
	Trust		How can we build public trust in the use of AI and BD technologies?
	Social acceptability		How to gain popular support for the use of intervention using AI and BD technologies?

### 3.1 Health outcomes and distribution

The literature is mostly speculative and ambivalent regarding AIS using BD capacity to generate positive health outcomes (Horvitz and Mulligan, 2015). The major threat of these systems may be the unfair distribution of these outcomes in the population and between populations.

#### 3.1.1 Uncertain outcomes

##### 3.1.1.1 Positive health outcomes

Many authors speculate that these technologies will create positive health outcomes for populations (Althobaiti, 2021; Cheng et al., 2020; Abramoff et al., 2021; Castagno and Khalifa, 2020; Kelly et al., 2020). Some of these positive expectations have been associated with specific optimization of various health services. Authors have mentioned the gain in terms of accessibility (Fornasier, 2019; Xie et al., 2020; Bates et al., 2018; Jones et al., 2020). The combined use of AI and BD opens a new scalability and the possibility to treat an unimaginable quantity of patients in comparison to what the actual workforce can offer (Abramoff et al., 2021). In that sense, AIS can offer a response to the actual health workers shortage that many health systems are facing. In parallel, these technologies could reduce the cost of health services (Kern et al., 2016; Grigorovich and Kontos, 2020) and make resource allocation more efficient (Stylianou and Talias, 2017; Galetsi et al., 2019; Sparrow and Hatherley, 2019; Bates et al., 2018; Machluf et al., 2017; Canaway et al., 2019; Peters and Buntrock, 2014). These benefits could be significant for low- and middle-income countries (LMICs) (Alami et al., 2020), where AIS could complement existing health services (Schwalbe and Wahl, 2020).

Authors have identified specific interventions that could be optimized with the integration of AI and BD such as helping to manage disease (Kerr et al., 2018), faster (Fornasier, 2019) and with more precision (Althobaiti, 2021), or otherwise facilitate diagnosis (Noorbakhsh-Sabet et al., 2019; Sparrow and Hatherley, 2019), determining appropriate treatments (Yang and Chen, 2018), e.g., with the use of precision medicine (Ahmed et al., 2020), and improving patient outcomes more generally (Canaway et al., 2019). Robots more specifically could help reduce loneliness (Miller, 2020) and otherwise induce positive emotions in older patients (Ienca et al., 2016), enhance their autonomy and thus reduce the burden on the healthcare system (de Graaf et al., 2015; Ienca et al., 2016). At the population level, AI and BD can support proactive interventions, particularly in populations of lower socioeconomic status (Machluf et al., 2017; Eng, 2004; Zhang et al., 2017), improve the prevention, prediction and treatment of chronic diseases (Lajonchere, 2018; Kern et al., 2016; Cool, 2016), make disease screening more efficient (Morgenstern et al., 2021), and facilitate epidemics surveillance (Galetsi et al., 2019; Cheng et al., 2020; Bates et al., 2018; Roberts, 2019) and the decision-making in cases of global health emergencies (Galetsi et al., 2019). AIS can offer more targeted populational interventions through so-called “precision public health” (Johnson, 2020; Dolley, 2018). Authors also noted benefits for healthcare systems including analyzing their inefficiencies (Manrique de Lara and Pelaez-Ballestas, 2020; Ho et al., 2020), detecting problems in health laboratories (Yang and Chen, 2018), facilitating the assessment of health technologies and drugs (Lajonchere, 2018) and streamlining the workflow (Thorpe and Gray, 2015a; Tang et al., 2018; Sparrow and Hatherley, 2019; Kostkova et al., 2016). Finally, the AI and BD technologies could optimize the research process at the very core of healthcare (Mooney and Pejaver, 2018; Sparrow and Hatherley, 2019; Balas et al., 2015; Joda et al., 2018) and facilitate the distribution of its benefits (Ahmed et al., 2020).

##### 3.1.1.2 Negative health outcomes

Conversely, authors have identified numerous negative health outcome that could be aggregated into two clusters. The first one focusses on the errors that could be introduced by AI and BD. System dysfunction or malfunction are part of the game (Satava, 2002) and an error in AIS used systemically in healthcare could lead to harming 1,000 of patients (Sparrow and Hatherley, 2019). There is a possibility of misdiagnosis because of bugs or the overreliance of healthcare professionals (HCP) on AIS (Morley et al., 1982). The efficiency of AIS can lead to lower the human scrutiny on the system and diminish human capacity to control the system (Sarbadhikari and Pradhan, 2020). Another risks is the use of an AIS for a purpose other than what it was designed for (Ahmed et al., 2020). In the same vein, the vulnerability of these systems for cyber-attacks could disrupt the use of AI devices and affect populations (Abdulkareem and Petersen, 2021). Errors do not only pertain to the systems and HCPs can misleadingly interpret the results (Stylianou and Talias, 2017; Satava, 2002), misleadingly interpret the results of AIS because of their reluctance or distrust AI predictions (Ahmed et al., 2020; Horgan and Ricciardi, 2017; Nebeker et al., 2019).

The second cluster highlights the reductionist view of health introduced by these systems and the risks that something important will be missed (Dolley, 2018). Careless use may lead to wrong results, harming populations (Alemayehu and Berger, 2016) and wasting resources (Green and Vogt, 2016). The central role of BD for AIS risk reducing populations to numbers, narrowing the whole human experience (Sparrow and Hatherley, 2019) characterized by, inter alia, its irrationality, unpredictability and vulnerability (Kerr et al., 2018; Brill et al., 2019; Ahmed et al., 2020; Delpierre and Kelly-Irving, 2018; Prainsack, 2020), as well as its cultural dimension, situatedness, and its reliance on values, preferences and beliefs (Mentis et al., 2018; Kee and Taylor-Robinson, 2020; van Deursen and Mossberger, 2018). This form of dehumanization (Althobaiti, 2021; Ienca et al., 2016) can be detrimental to the therapeutic relationship (Sparrow and Hatherley, 2019) by providing services devoid of human contact (Fulmer, 2019; Kernaghan, 2014; Miller, 2020; Cordeiro, 2021), the empathy and the compassion normally offered by HCP (Kerr et al., 2018; Morley et al., 1982; Manrique de Lara and Pelaez-Ballestas, 2020). Also, these technologies are seen as ways to ground the personalization of medicine based on the individual’s genetic background. For some, it is feared that this narrow use will divert the focus away from public health interventions, and from upstream determinants of health (Kee and Taylor-Robinson, 2020; Kenney and Mamo, 2019).

#### 3.1.2 Fair distribution of the outcomes

In parallel to the ambivalent outcomes of AIS for population health, many authors suggest that a central issue of these technologies will be to the inequitable distribution of their outcomes (Vollmer Dahlke and Ory, 2020; Althobaiti, 2021; Ienca et al., 2016; Conway, 2014; Ossorio, 2014; Rosen et al., 2020; Samuel and Derrick, 2020; Terrasse et al., 2019; Amann et al., 2020; Xafis et al., 2019; Car et al., 2019). They fear that these technologies’ health benefits will be concentrated in the hands of the more privileged groups while the burdens will be transferred to the less privileged. Five areas of reflections regarding the fair distribution have been scrutinized.

##### 3.1.2.1 Increase of health disparities

Because of the scale at which it is used (Abramoff et al., 2021), some hope that AIS used in population health interventions will contribute to reducing health disparities (Zhang et al., 2017; Genevieve et al., 2019), but the reverse effect is anticipated by many (Nebeker et al., 2019; Terrasse et al., 2019; Breen et al., 2019; Holzmeyer, 2021; Luk et al., 2021). Some fear that these technologies will affect disproportionately parts of the population (Zhang et al., 2017; Montgomery et al., 2018) such as people with disabilities (Jones et al., 2020), vulnerable populations (Rosen et al., 2020), and marginalized communities (Hu et al., 2017). This could be partly due to interventions (e.g., precision public health) narrowly focused on biomedical factors and surveillance instead of taking into consideration social determinants of health (Johnson, 2020; Mentis et al., 2018; Kenney and Mamo, 2019; Backholer et al., 2021; Trein and Wagner, 2021). Conversely, public health surveillance programs may unduly focus on vulnerable populations because they may have less control over their “digital footprint” (Rosen et al., 2020), be insufficiently prepared to represent their interests (van Deursen and Mossberger, 2018) and lack time to manage their virtual identity (Montgomery et al., 2018). In parallel, there is a risk that the technology be used with bad intentions, perpetuating social prejudices and therefore increase health disparities (Abdulkareem and Petersen, 2021). For example, discriminatory uses of BD and AIS, such as selecting who has access to healthcare (Sparrow and Hatherley, 2019), identifying noncompliant patients (Moutel et al., 2018) and cherry-picking patients (Zhang et al., 2017; Terrasse et al., 2019), could increase health disparities by depriving populations who need it most from access to health services (Cahan et al., 2019).

##### 3.1.2.2 Discrimination and stigmatization

Another type of justice consideration regarding BD and AIS relates to discrimination and stigmatization. Data breaches; loss of privacy; public information on social media; the identification of individuals, falsely or not, with a medical condition, a particular genotype, or as the source of an infection (Raza and Luheshi, 2016; Shachar et al., 2020); and the inclusion of social determinants in electronic health records (Goodman, 2020) and tracing apps (Mbunge, 2020); all these situations raise risks of stigmatizing individuals and communities (Aiello et al., 2020; Galetsi et al., 2019; Hunt et al., 2020; Genevieve et al., 2019; Breen et al., 2019; Luk et al., 2021; Baldassarre et al., 2020; Mikal et al., 2016; Vayena et al., 2015; Yang and Chen, 2018; Celedonia et al., 2021; Ngan and Kelmenson, 2021; Straw, 2021; Xing et al., 2021) as well as risks of discrimination (Cordeiro, 2021; Salerno et al., 2017; Yeung, 2018; Gilbert et al., 2019; Chen and See, 2020; Jalal et al., 2020) by insurance companies and employers (Stylianou and Talias, 2017; Cahan et al., 2019; Docherty and Lone, 2015; Colloc, 2015; Salas-Vega et al., 2015; Benke and Benke, 2018; Sparrow and Hatherley, 2019; Brill et al., 2019; van Deursen and Mossberger, 2018; Manrique de Lara and Pelaez-Ballestas, 2020; Montgomery et al., 2018; Salerno et al., 2017; Sun et al., 2020; Babyar, 2019; Ajunwa et al., 2016; Adkins, 2017; Casanovas et al., 2017; Ienca et al., 2018; Mootz et al., 2020; Tigard, 2019; Rajam, 2020). These risks apply even to individuals who have not participated in research activities (Kim et al., 2017) (e.g., when members of a group have shared identifiers) (Manrique de Lara and Pelaez-Ballestas, 2020) and when data has been anonymized (Dolley, 2018; Sanchez M and Sarria-Santamera, 2019). Discrimination could also occur on the basis of race, sex (Abdulkareem and Petersen, 2021; Manrique de Lara and Pelaez-Ballestas, 2020), gender (Zou and Schiebinger, 2021), income, age (Abramoff et al., 2021), and it can take many forms such as “invisibility, exclusion, or complacency employed to avoid detection, critique, or questioning” (Dankwa-Mullan et al., 2021). At the clinical level, protocols based on population statistics may exclude the individual preferences of patients (Dagi, 2017).

##### 3.1.2.3 Digital colonialism

One distribution consideration relates to the fair return of results of technology development. Authors highlight the risk of “digital colonialism” where privileged populations benefit from the development of technology while the less privileged are left apart. This issue can take many forms that are mostly illustrated by the unequal relationships between high-income countries and LMICs. One fear is that researchers from high-income countries take advantage of data collected by researchers in LMICs for their own advantage and without acknowledging the latter’s work (Ballantyne, 2019; Car et al., 2019; Howe and Elenberg, 2020). At the population level, some worry that health data be analyzed in high-income settings with no possibility for LMIC to control how it is used (Andanda, 2019) and to benefit from it (Dolley, 2018; Li and Cong, 2021). Digital colonialism can also take the form of AIS being developed with data from high-income countries that will lead to detrimental and discriminatory effects on health care in LMICs. For example, these AIS may recommend a health intervention that is not feasible locally or only available at a significative costs outside the country (Alami et al., 2020; Bhattacharya et al., 2021; Demuro et al., 2020). Another consideration is that there might be socioeconomical barriers that prevent the implementation, in a LMIC, of an algorithm created in a high-income country (Liu and Bressler, 2020). A corollary is “ethics dumping,” which is “exporting unethical research practices, for example, unethical data processing […] to countries where research ethics committee oversight is lacking” (Samuel and Derrick, 2020). Some could justify this “ethics dumping” with the fact that the access to healthcare can be difficult in some LMICs. In the same vein, there is a concern that non-compliant technologies could bypass security and privacy vulnerabilities since informal healthcare is more prevalent in LMICs countries (Alami et al., 2020). However, this could lead us to a new “medicine for the poor” in the same way that most of the medical equipment being sent to LMICs fail or do not work (Alami et al., 2020).

##### 3.1.2.4 Digital divide

The “digital divide” argument offers a variation on the unfair distribution of outcomes issue (Zhang et al., 2017). It describes inequalities in access to data (van Heerden et al., 2020) and technologies (Bennett, 2019) caused either by of a lack of resources (Mbunge, 2020) or knowledge (Aiello et al., 2020; Galetsi et al., 2019; Bennett, 2019; Vollmer Dahlke and Ory, 2020; Genevieve et al., 2019; Lodders and Paterson, 2020). The increased use of BD and AI in health could worsen the digital divide (Eng, 2004) and perpetuate health inequities (Genevieve et al., 2019; Murphy et al., 2021) by leaving out people who cannot or do not want to use those technologies (Kerr et al., 2018; Cordeiro, 2021; Mootz et al., 2020; Fleming, 2021). This can particularly affect people in LMICs (Brill et al., 2019; Mentis et al., 2018; Manrique de Lara and Pelaez-Ballestas, 2020; Mbunge, 2020) but also populations with lower socioeconomic status in high-income countries (Budd et al., 2020). The digital divide could have multiple consequences. First, it could lead to unrepresentative data sets by excluding populations who have least access to technologies (Cahan et al., 2019; Docherty and Lone, 2015; Benke and Benke, 2018; Aiello et al., 2020; Vollmer Dahlke and Ory, 2020; Dolley, 2018; Delpierre and Kelly-Irving, 2018; Ossorio, 2014; Genevieve et al., 2019; Breen et al., 2019; Mikal et al., 2016; Yeung, 2018; Gilbert et al., 2019; Hodgson et al., 2020). Second, these populations have higher burdens of disease (e.g., advanced age, lower economic status, etc.) but have less resources to benefit from BD and AI innovations (Larkin and Hystad, 2017; Sun et al., 2020; Strang, 2020). Third, the digital divide could also create inequities in digital surveillance (Aiello et al., 2020) and be exacerbated by the uses of the technologies at the international level (Manrique de Lara and Pelaez-Ballestas, 2020). However, populations with lower digital literacy could also be overrepresented because they “may be more likely to unknowingly imply consent” (Mikal et al., 2016; Demuro et al., 2020). Programs aiming to curb the digital divide could create a “privacy divide” if they require that vulnerable populations trade their personal data in exchange for products and services (Montgomery et al., 2018).

##### 3.1.2.5 Biases in datasets and algorithms

An important concern relates to the presence of biases in datasets and the coding of algorithms that may lead to an unfair distribution of the benefits and burdens of the technology in the population or between populations. Biases may have different sources such as the obliteration of certain groups in the datasets used to train AI. This could come from observational, and sampling bias at the basis of data gathering (Cahan et al., 2019; Howe and Elenberg, 2020; Bhattacharya et al., 2021; Strang, 2020; Goldsmith et al., 2021; Tan et al., 2020) or missing data from less represented populations (Cahan et al., 2019; Lanier et al., 2020; Howe and Elenberg, 2020; Bhattacharya et al., 2021; Strang, 2020; Goldsmith et al., 2021; Tan et al., 2020). Biases in programming (Cahan et al., 2019; Wang and Alexander, 2020; Xie et al., 2020; Ahmed et al., 2020), for its part, may come from the amplification of previous biases and the failure to recognize them in subsequent stages (Morgenstern et al., 2021; Zou and Schiebinger, 2021; Baclic et al., 2020; Thomasian et al., 2021). They could also come from the erroneous decision to apply data from one population to another (Tang et al., 2018; Sparrow and Hatherley, 2019; Zhang et al., 2017; Morley et al., 1982; Delpierre and Kelly-Irving, 2018) or developers’ incorrect assumptions and beliefs (Terrasse et al., 2019; Yeung, 2018). All this will result in biased results, or to what authors refer to with the expression “garbage in, garbage out” (GIGO), meaning that biased data leads to biased results (Cahan et al., 2019; Howe and Elenberg, 2020; Evans et al., 2020). The risk at this stage is the perpetuation of biases, as biased algorithms could exacerbate already present racial and socioeconomic inequalities and vulnerabilities (Sarbadhikari and Pradhan, 2020; Luk et al., 2021; Couch et al., 2020). This may affect the health of individual patients (Tang et al., 2018; Morley et al., 1982; Sarbadhikari and Pradhan, 2020) and, moreover, the wellbeing of the global population (Docherty and Lone, 2015; Galetsi et al., 2019; Breen et al., 2019; Yeung, 2018) in terms of the perpetuation of discriminatory racial and social practices (Cahan et al., 2019; Kee and Taylor-Robinson, 2020; van Deursen and Mossberger, 2018; Altenburger and Ho, 2019) or health inequities (Cahan et al., 2019; Lanier et al., 2020; Sparrow and Hatherley, 2019; Zhang et al., 2017; Manrique de Lara and Pelaez-Ballestas, 2020; Yeung, 2018; Ballantyne, 2019; Villongco and Khan, 2020; Carney and Kong, 2017). Biased AIs seem unavoidable (Goodman, 2020), or hard to minimize (Gilbert et al., 2019), because of the black-box nature of many AIS (Lanier et al., 2020; Sparrow and Hatherley, 2019; Rajam, 2020), and the ubiquitous nature of AI (van Deursen and Mossberger, 2018). The mistake may be to consider data as pure and objective realities (Holzmeyer, 2021) although they are determined (like health and wellbeing) by economic, social, and political dynamics (Johnson, 2020; Delpierre and Kelly-Irving, 2018) as well as generating social consequences (Alami et al., 2020).

### 3.2 Health determinants

Aside from discussing the health outcomes and their distribution in the population and between populations, the literature reflects on how AIS and BD will affect three important health determinants: health behaviors, healthcare functioning, and data infrastructure.

#### 3.2.1 Promotion of healthy behaviors

Looking at how the technologies will affect health-related behaviors, the literature is dubious by both acknowledging their potential for individual empowerment as well as their possibility to undermine the individuals’ autonomy (Snell, 2019). Digital literacy appears to be an important condition to obtain such positive outcomes.

##### 3.2.1.1 Empowerment and disempowerment

AIS using BD may affect positively individual behaviors by empowering patients in taking care of their own health (Fornasier, 2019; Lajonchere, 2018; Fulmer, 2019; Cordeiro, 2021; Manrique de Lara and Pelaez-Ballestas, 2020; Snell, 2019; Montgomery et al., 2018; Prosperi et al., 2018). These technologies could help individuals to monitor their own health (Wang and Alexander, 2020; Kenney and Mamo, 2019; Car et al., 2019), offer pertinent health information (Prosperi et al., 2018; Kostkova et al., 2016), contribute to decision-making (Bellazzi, 2014; van Deursen and Mossberger, 2018), assist in the management of their health and illness (Eng, 2004), and open the possibility of robotic assistance and interactions (Bennett, 2019; de Graaf et al., 2015; Vollmer Dahlke and Ory, 2020; Belk, 2020; Kernaghan, 2014). All this could be of great use for chronic disease management (Kenney and Mamo, 2019; Car et al., 2019), and supporting disabled people (Jones et al., 2020) or elderly people’s autonomy (Manzeschke et al., 2016). The autonomy offered by these systems may modify the power relationship with the HCP in favor of the patient (Galetsi et al., 2019; Sparrow and Hatherley, 2019; Terrasse et al., 2019; Gilbert et al., 2019), thus diminishing medical authority (Lupton and Jutel, 2015). This conception of empowerment and engagement is a strong dimension of the digital health rhetoric (Lupton and Jutel, 2015).

The combined use AI and BD can also have positive effects on collective behaviors. Some anticipate that these technologies offer platforms for collective engagement, for example in disease surveillance (Genevieve et al., 2019; Sun et al., 2020) and in the research process (Manrique de Lara and Pelaez-Ballestas, 2020; Manrique de Lara and Pelaez-Ballestas, 2020; Gossec et al., 2020; Anisetti et al., 2018; Katapally, 2020). In that vein, some see the possibility of citizen engagement in the development of these very same technologies (Casanovas et al., 2017; Altenburger and Ho, 2019), yet a lot has still to be done (Conrad et al., 2020; Breen et al., 2019; Gilbert et al., 2019; Pepin et al., 2020; Nichol et al., 2021; Tang et al., 2018; Manzeschke et al., 2016). This possibility raises its very own ethical issues regarding the authenticity of the engagement of citizens, patients or populations (Bennett, 2019; Vollmer Dahlke and Ory, 2020; Ballantyne, 2019; Evans et al., 2020).

Conversely, many speculate that the technologies will promote disempowerment. For some, patients may feel a loss of agency toward the decision taken by HCPs and AIS (Morley et al., 1982; van Deursen and Mossberger, 2018), particularly if the AIS is opaque (Amann et al., 2020), and create forms of nudging (van Deursen and Mossberger, 2018). Aside, the pervasiveness of the technology may discourage individuals to engage in their own health and leave this task to the technology (Kasperbauer, 2021). On the other hand, they may feel responsible for their health, creating “individuals on alert” (van Deursen and Mossberger, 2018; Samerski, 2018). Despite presenting themselves as patient-empowering, self-diagnosis apps still recommend users to seek medical advice, challenging patient empowerment in face of medical authority (Lupton and Jutel, 2015).

##### 3.2.1.2 Digital and ethical literacy

To sustain the empowering of populations and attain positive health outcomes, digital and ethical literacy appears to be an essential precondition for the stakeholders of population health (Benke and Benke, 2018; Fulmer, 2019; Delpierre and Kelly-Irving, 2018; Ballantyne, 2019). First, there is a need to educate the public regarding digital technologies using BD and AI (Galetsi et al., 2019; van Deursen and Mossberger, 2018) and their various pitfalls such the limitations of the technology (Grigorovich and Kontos, 2020; van Deursen and Mossberger, 2018; Lupton and Jutel, 2015), the complexity of privacy protection (Mikal et al., 2016; Lodders and Paterson, 2020), the risks of cybersecurity (van Deursen and Mossberger, 2018) and the inherent biases of the technology (Mikal et al., 2016). The same necessity for digital and ethical literacy by the general population has been said for policymakers (Lanier et al., 2020), HCP, and researchers (Stylianou and Talias, 2017; Leyens et al., 2017; Aiello et al., 2020; Galetsi et al., 2019; Brill et al., 2019; Machluf et al., 2017; Satava, 2002; Babyar, 2019; Gossec et al., 2020; Pepin et al., 2020; Hemingway et al., 2018; Godfrey et al., 2020; Ho and Caals, 2021). At last, ethical literacy may be critical for data scientists to achieve their aim (Dolley, 2018; Kern et al., 2016; Zhang et al., 2017; van Deursen and Mossberger, 2018; Goodman, 2020; Nebeker et al., 2019).

#### 3.2.2 Efficient healthcare functioning

Aside from health behaviors, authors have dissected the effects of AIS on more structural health determinants such as healthcare accessibility and quality. Regarding that pattern of determinants, it is anticipated that AIS will transform healthcare working conditions (Noorbakhsh-Sabet et al., 2019). Some speculate the potential of the technologies to maximize HCPs’ workforce, others suggest an increased workload and a devaluation of their work.

On the positive side AIS could assist HCPs in their work through numerous tasks such as removing repetitive tasks (Ahmed et al., 2020; Jalal et al., 2020), improving workflow (Tang et al., 2018), managing patients (Kerr et al., 2018; Pagliari, 2021), keeping pace with the medical literature (Conrad et al., 2020), supporting diagnostic and treatment decisions (Benke and Benke, 2018; Jones et al., 2020; Adkins, 2017; Wang et al., 2021), personalizing treatment (Brill et al., 2019), and possibly even reducing misdiagnosis (Xie et al., 2020). They could also support communication between HCPs and patients, maximizing the short time given for clinical consultations (Mootz et al., 2020). Thus, AIS, instead of dehumanizing care, would help rehumanize (Cahan et al., 2019; Conrad et al., 2020; Belk, 2020) and reinforce the relationship (Brill et al., 2019).

There is no consensus on the potential benefits of AIS. Many fear an increase in HCPs’ workload (Grigorovich and Kontos, 2020; Xing et al., 2021). The necessity for HCPs to adapt to new AIS by learning how to use the technology (Kerr et al., 2018; Grigorovich and Kontos, 2020; Godfrey et al., 2020) and the incentive to collect and manage more data, will all add to their workload (Sparrow and Hatherley, 2019; Grigorovich and Kontos, 2020). For example, electronic-health records add administrative burdens for HCPs (Sparrow and Hatherley, 2019). Also, the optimization of the services may lead to treat more patients instead of allowing more time for clinical consultations (Sparrow and Hatherley, 2019). Furthermore, the use of apps for health self-monitoring may lead to increased and unnecessary referrals to HCPs, also adding to their workload (Ienca et al., 2018).

In the long term, many authors raise the concern that AIS could change the healthcare workforce (Benke and Benke, 2018) by devaluating their expertise (Kasperbauer, 2021). Some anticipate that doctors (Stylianou and Talias, 2017; Galetsi et al., 2019; Fulmer, 2019; Ahmed et al., 2020; Satava, 2002; Manrique de Lara and Pelaez-Ballestas, 2020; Conrad et al., 2020; Conrad et al., 2020; Samerski, 2018), and nurses (Goodman, 2020) be replaced by AIS (Althobaiti, 2021), although it is not unanimously supported (Cahan et al., 2019; Lajonchere, 2018; Tang et al., 2018; Adkins, 2017; Belk, 2020). The replacement of HCP by AIS could lead to diminished professional autonomy (Lajonchere, 2018; Sparrow and Hatherley, 2019; Dagi, 2017), dependence to AIS (Abramoff et al., 2021), deskilling of HCPs (Sparrow and Hatherley, 2019; Satava, 2002; Goodman, 2020), and unemployment (Kerr et al., 2018). Authors also highlighted the risk of increasing the surveillance of workers (Tang et al., 2018; Sparrow and Hatherley, 2019; Yang and Chen, 2018).

#### 3.2.3 Data control

From a perspective of health technologies more and more dependent of big data, the control over data plays a strategical role. Who controls data may direct the benefits downstream and affect the health of entire populations. For that reason, issues of data control shape a specific pattern of health determinants. In relation to that concern, three groups of issues play a preponderant role in the literature: issues over data ownership, data management, and data accessibility.

##### 3.2.3.1 Data ownership

The question of data ownership asks the question of who can exercise power over the data that will be used to train and fed AI. The question of ownership is a complex one (Gilbert et al., 2019). Data are created by many people and all have some rights over the data (Mooney and Pejaver, 2018; Tang et al., 2018) while promoting different agendas (Alemayehu and Berger, 2016). BD derived technologies amplify this situation with their capacity, sometime furtive, to aggregate numerous sources of data. These sources of data may be as diverse as ordinary internet-connected object (Miller, 2020; van Deursen and Mossberger, 2018; Ajunwa et al., 2016) to public health surveillance interventions (Hodgson et al., 2020). All this may be complicated by the limited knowledge of the data individuals implicitly share (Horvitz and Mulligan, 2015).

A strong line of thought suggests that there is an information asymmetry between individual and corporation in the favor of the latter (Sun et al., 2020). Health data can be seen as a profitable investment for corporations (Canaway et al., 2019; Cheung, 2020). There is the possibility that private corporation owns sensitive health information (Tigard, 2019) and that they capture health data coming from public health interventions (Aiello et al., 2020). Although they might be regulated (Ballantyne, 2019), they might be less accountable for the use of data (Andanda, 2019) while caring less for the social good (Terrasse et al., 2019) than the protection of intellectual property (Salas-Vega et al., 2015) and developing monopolies (Ladner and Ben Abdelaziz, 2018; Roberts, 2019; Satava, 2003). This situation opens fear of abuses (Benke and Benke, 2018; Delpierre and Kelly-Irving, 2018; Prainsack, 2020) which makes some believe that the deployment of AIS will benefit the corporation rather than the populations (Adkins, 2017) and perpetuate social inequalities (Andanda, 2019).

While corporations play a central role in data economies, the control of individuals over their own data also need to be considered (Andanda, 2019; Colloc, 2015; Salas-Vega et al., 2015; Benke and Benke, 2018; Tang et al., 2018). Policies may play an important role in protecting this form of control (Montgomery et al., 2018) to respond to constant risk of reidentification (Delpierre and Kelly-Irving, 2018) and commodification (Conrad et al., 2020). Traditionally, patients have not been able to control their healthcare data (Bates et al., 2018), but, because of the strategic role data plays. There is an increasing demand from individuals to have access to their own data (Stylianou and Talias, 2017; Hodgson et al., 2020).

An alternative to previous mode of property could be find in collective ownership of data such as “data sovereignty” which could be defined as the “rights of a nation to govern the collection, ownership and use of its own data” (Ballantyne, 2019). It is argued that people using AIS should have the chance to have some control over their data (Cordeiro, 2021; Casanovas et al., 2017), particularly if we consider that the data provided for the development of BD and AIS is a community investment (Oravec, 2019). As a community investment, it may warrant financial returns or a stake in the decision-making (Oravec, 2019). Differences in data systems between countries raise challenges and opportunities for State-bodies (Machluf et al., 2017). Governmental control can be seen as a more secure (Snell, 2019) alternative to commercial management. Governance innovations include “data custodians and/or indigenous data governance bodies” (Ballantyne, 2019). This control of data by communities may contribute to guarantee the inclusion of diverse dimensions and include social determinants of health (Dankwa-Mullan et al., 2021). Although, this community control may be illusory if, at the end, data are stored in the cloud through a network of foreign servers (Colloc, 2015).

##### 3.2.3.2 Data management

A related set of issues to the ones of ownership relates to data management (Stylianou and Talias, 2017; Salas-Vega et al., 2015; Galetsi et al., 2019; Wang and Alexander, 2020; Ladner and Ben Abdelaziz, 2018; Ahmed et al., 2020; Young, 2018). Authors ask what ethical data management would look like (Samuel and Derrick, 2020)? Data management is an important consideration because of the increasing number of people involved in data collection (Vogel et al., 2019), and the enormous amount of data generated (Bellazzi, 2014). Data management implies a long continuum from data production, storage, curation, analysis, protection and circulation. It raises the issue of who has the power to manage the data and the risk of centralized or commercial data control (Manrique de Lara and Pelaez-Ballestas, 2020; Tupasela et al., 2020). On the contrary, centralization may be replaced by over fragmentation and make it difficult to locate when data are used as part of large platforms or by many entities, e.g., in research settings (O’Doherty et al., 2016). In terms of population health, the more acute concern is to optimize their use (Cahan et al., 2019), because of their medical importance (Casanovas et al., 2017), and their role in eliminating health disparities (Carney and Kong, 2017). Authors sometimes talk about the stewardship of data (Sanchez M and Sarria-Santamera, 2019) which includes the “safeguards, audits and operational protocols” (Sanchez M and Sarria-Santamera, 2019).

One risk associated with data management are conflicts of interests (COI) that can arise if data belong to actors who have diverging interests. For example, corporations, governments, the public, healthcare systems, HCPs and researchers may all have diverging needs, interests and goals, raising risks of COI (Salas-Vega et al., 2015; van Deursen and Mossberger, 2018; Manrique de Lara and Pelaez-Ballestas, 2020; Salerno et al., 2017; Casanovas et al., 2017; Car et al., 2019; Altenburger and Ho, 2019). This situation may be more patent for regulators who want to promote, at the same time, commercial and public interests (Tupasela et al., 2020). At last, COIs can be hidden within the programming of their algorithms (Car et al., 2019).

##### 3.2.3.3 Data accessibility and sharing

Corollary issues regard the accessibility of the data and data sharing (Stylianou and Talias, 2017; Wang and Alexander, 2020; Benke and Benke, 2018; Galetsi et al., 2019; Ladner and Ben Abdelaziz, 2018; Ahmed et al., 2020; Nebeker et al., 2019; Goodman, 2020; Sun et al., 2020; Ienca et al., 2018; Tigard, 2019; Hodgson et al., 2020). Publicly funded data and data of public utility may have a stronger obligation for being accessible (Tang et al., 2018; Goodman, 2020; Gossec et al., 2020). Access to data is necessary in order to realize BD and AI’s potential for improving global (Li and Cong, 2021; Galetsi et al., 2019) and individual health (Zhang et al., 2017; Kostkova et al., 2016; Raza and Luheshi, 2016; Li and Cong, 2021; Mahlmann et al., 2017). The accessibility of data can be essential for public health, and become critical during infectious outbreaks and (Budd et al., 2020; Ballantyne, 2019). Data sharing is also strategic for research activities (Galetsi et al., 2019; Zhang et al., 2017). For example, easier access to publicly funded clinical datasets could help reduce data-access inequities between researchers (Zhang et al., 2017), and enable reproducible research (Gossec et al., 2020).

Because of its benefits, some people believe that individuals have a duty to share their data in order to advance health goals. Some authors defend the idea that it is a societal responsibility to act accordingly (Green and Vogt, 2016). In other words, individuals have a duty to share their information for the sake of their own treatment (Salerno et al., 2017), for epidemiological reasons (Salerno et al., 2017), for the advancement of health research (Tsai and Junod, 2018) or for the learning health system (Sparrow and Hatherley, 2019). Individuals will benefit at a certain point in life from these goods (Tsai and Junod, 2018) or they will contribute to the common good (Snell, 2019). Otherwise, it may be considered as selfishness or free riding (Snell, 2019).

However, this imperative to share data may face several barriers that may be practical (Leyens et al., 2017; Wang and Alexander, 2020; Ahmed et al., 2020; Balas et al., 2015; Lee and Yoon, 2017), cultural (Leyens et al., 2017; Tang et al., 2018; Car et al., 2019), economical (Tang et al., 2018; Lee and Yoon, 2017; Machluf et al., 2017), technical (Lajonchere, 2018; Noorbakhsh-Sabet et al., 2019; Zhang et al., 2017; Raza and Luheshi, 2016; Yang and Chen, 2018; Dagi, 2017; Hemingway et al., 2018; Salerno et al., 2017; Deshpande et al., 2019), political (Machluf et al., 2017; Car et al., 2019; Carney and Kong, 2017), ethical (Bates et al., 2018; Machluf et al., 2017; Balas et al., 2015; Yang and Chen, 2018; Gilbert et al., 2019; Prosperi et al., 2018; van Heerden et al., 2020), and regulatory (Salas-Vega et al., 2015; Lajonchere, 2018; Zhang et al., 2017; Manrique de Lara and Pelaez-Ballestas, 2020; Rosen et al., 2020; Raza and Luheshi, 2016; Yang and Chen, 2018; Casanovas et al., 2017; Mahlmann et al., 2017; Deshpande et al., 2019). Many stakeholders may have an interest in accessing data, e.g., researchers, health-policy makers, HCPs, insurances (Stylianou and Talias, 2017). It raises numerous questions. Who should be given access to the data? For which aim? In which conditions? (Stylianou and Talias, 2017; Casanovas et al., 2017) With which safeguards? (Andanda, 2019; Lajonchere, 2018) In which sustainable infrastructure? (Raza and Luheshi, 2016; Pepin et al., 2020) How should benefits and risks of data sharing should be distributed equitably? (Brill et al., 2019; Ballantyne, 2019) These questions are entangled in the web of issues at the intersection of privacy protection, control over data access, and protecting informed consent (Sanchez M and Sarria-Santamera, 2019).

### 3.3 Interventions and policies

So far, we have seen the ethical tension raised by AIS and their reliance on BD from the perspective of their effect on health outcomes and patterns of health determinants. For this last part, we will look at their effect on intervention and policies. Intervention and policies are seen as ways to work on health determinants to produce greater health outcomes for the population. Looking at the means of population health, the discussion may be summarized as how the uses of the technologies may infringe common ethical and legal obligations in terms of privacy, consent, responsibility, transparency, trust and social acceptability.

#### 3.3.1 Privacy protection

Privacy could be defined as “the right to be left alone” (Ienca et al., 2016). Sun and collaborators argue that, in the context of health, privacy refers to one’s right to decide what identifiable data is collected, how it is used and disclosed (Sun et al., 2020). AIS and BD in population health raise various multidimensional privacy issues (Bellazzi, 2014; Benke and Benke, 2018; Tang et al., 2018; Kerr et al., 2018; Noorbakhsh-Sabet et al., 2019; Althobaiti, 2021; Abramoff et al., 2021; Ahmed et al., 2020; van Deursen and Mossberger, 2018; Cordeiro, 2021; Conway, 2014; Gilbert et al., 2019; Prosperi et al., 2018; Pepin et al., 2020; Hemingway et al., 2018; Joda et al., 2018; Kayaalp, 2018; Shahid et al., 2021) which are seen as an important concern for the public (Mooney and Pejaver, 2018; Comess et al., 2020) and HCPs (Castagno and Khalifa, 2020) because of the significant importance of health data (Heitmueller et al., 2014). However, as we will see, empirical data may mitigate the importance accorded by the public to privacy issues (Esmaeilzadeh, 2020). Two dimensions are of particular interest for ethics: privacy breaches and the difficult operationalization of privacy standards.

##### 3.3.1.1 Privacy breaches

Privacy issues are central to the ethics of AIS and BD because of the informational nature of these technologies. They refer mostly to wrongful uses of data (Colloc, 2015; Balas et al., 2015; Shah and Khan, 2020), accidental disclosure (Mooney and Pejaver, 2018; Balas et al., 2015; Conway, 2014; Casanovas et al., 2017), data crossing (Sparrow and Hatherley, 2019; Horvitz and Mulligan, 2015) or unintentional disclosure of sensitive information (Xing et al., 2021; Murphy et al., 2021; Gilbert et al., 2020). These are frequently analyzed through the lens of cybersecurity issues (Stylianou and Talias, 2017; Tanti, 2015; Dolley, 2018; Liyanage et al., 2014; Lajonchere, 2018; Salas-Vega et al., 2015; Wang and Alexander, 2020; Heitmueller et al., 2014; Kerr et al., 2018; Xie et al., 2020; Galetsi et al., 2019; Sparrow and Hatherley, 2019; Fulmer, 2019; Althobaiti, 2021; Bates et al., 2018; Machluf et al., 2017; Ahmed et al., 2020; Canaway et al., 2019; Ienca et al., 2016; Kostkova et al., 2016; Balas et al., 2015; Prainsack, 2020; van Deursen and Mossberger, 2018; Ossorio, 2014; Rosen et al., 2020; Montgomery et al., 2018; Salerno et al., 2017; Gilbert et al., 2019; Sun et al., 2020; Ajunwa et al., 2016; Casanovas et al., 2017; Ienca et al., 2018; Mootz et al., 2020; Tigard, 2019; Dagi, 2017; Hodgson et al., 2020; Lupton and Jutel, 2015; Pepin et al., 2020; Manzeschke et al., 2016; Belk, 2020; Snell, 2019; O’Doherty et al., 2016; Deshpande et al., 2019; van Heerden et al., 2020; Cutrona et al., 2012; Fornasier, 2019; Hoffman and Podgurski, 2013; Terry, 2014; Torous and Haim, 2018; Tsai and Junod, 2018; Veiga and Ward, 2016). Privacy breaches are increasingly observed in the health sector (Sun et al., 2020; Ajunwa et al., 2016; Dagi, 2017) and they have been highlighted at different phases of health data circulation from collecting (Mohr et al., 2017), transferring between linked services (Shah and Khan, 2020), sharing (Salerno et al., 2017), storing (Stylianou and Talias, 2017; Larkin and Hystad, 2017; Kostkova et al., 2016; Conway, 2014; Gossec et al., 2020; O’Doherty et al., 2016), training AIS (Murphy et al., 2021) to destructing data (Wang et al., 2021).

The main harm of privacy breaches may be the risks of re-identification. Even if data are anonymized, many studies have shown that individuals can often be re-identified (Lee et al., 2016). Re-identification can be done by linking anonymous data, meta data (Gilbert et al., 2019) and datasets (Docherty and Lone, 2015) and is made easier with interoperable datasets (Delpierre and Kelly-Irving, 2018). Many authors in this review agree that re-identification risks are high with BD and related technologies. The re-identification risk increases with data’s dimensionality, i.e., the number of variables of data (e.g., age, location, weight, any other physiological trait, genetic information, etc.) (Bellazzi, 2014; Cahan et al., 2019; Mooney and Pejaver, 2018). Re-identification risks also increase with the low prevalence of the variable (e.g., rare medical conditions) (Docherty and Lone, 2015; Ballantyne, 2019; Demuro et al., 2020), the quantity of personal data in the public domain (Tsai and Junod, 2018), data linkage (Manrique de Lara and Pelaez-Ballestas, 2020), combination of data (Rennie et al., 2020), the improvement of data mining methods (Lee et al., 2016), and who has access to it, at the end, creating various degrees of de-identification (Ballantyne, 2019).

Surveillance activities raise particular concerns in terms of privacy. They are troubling considering the staggering amounts of data held by health organizations, corporations (Celedonia et al., 2021; Lodders and Paterson, 2020) and governments that can be used against the interest of individuals (Andanda, 2019; Baldassarre et al., 2020; Evans et al., 2020). The risk of surveillance is an unavoidable trade-off of the of BD (Ngan and Kelmenson, 2021; Howe and Elenberg, 2020) and AIS in health-related activities and one that attenuates its possible benefits (Galetsi et al., 2019; Mootz et al., 2020). For example, passive technologies such as imbedded sensors are less intrusive than direct observation (Grigorovich and Kontos, 2020), but nonetheless imply the collection of immense quantities of data. In the context of the COVID-19 pandemic, citizen populations were watched in order to prevent the spread of the disease, but many expressed concerns that this information be used for other purposes (Sarbadhikari and Pradhan, 2020; Shachar et al., 2020; Naudé, 2020; Shen and Wang, 2021).

##### 3.3.1.2 Operationalization of privacy standards

The operationalization of privacy standards faces several challenges. It is not clear how to use the polysemic concept of privacy (Mooney and Pejaver, 2018; Conway, 2014; Casanovas et al., 2017; Snell, 2019). Some suggest to distinguish different forms of privacy, which certain forms are more at risk with BD such as informational privacy (Ienca et al., 2016) or physical privacy through surveillance (Bennett, 2019). The complexity may also arise because of the overlapping of privacy with a large spectrum of ethical values such as trust, transparency, security and property over who has access to the data and for what uses (Canaway et al., 2019). Contexts may also influence the definition and operationalization of privacy. For example, different areas of research have various methodologies and tools, complicating the protection of privacy in interdisciplinary health research (Casanovas et al., 2017).

Culture could also influence how privacy is understood, raising the question of whether a core definition should be used across all settings or not (Vayena et al., 2015). Also, in some political and economic contexts, citizens may consider that privacy concerns are irrelevant because of the level of surveillance already imposed by the State (Liu and Graham, 2021). Authors also note regularly the paradox between the perceived lack of concern of people toward sharing identifiable information on internet platforms (Aiello et al., 2020; Mikal et al., 2016; Yang and Chen, 2018; Snell, 2019; Young, 2018) and, at the same time, the fear of privacy breach related to participation in research project (de Graaf et al., 2015; Montgomery et al., 2018; Mootz et al., 2020; Tsai and Junod, 2018; Wongkoblap et al., 2017), public health interventions (O’Doherty et al., 2016) or any other health activities (Yang and Chen, 2018).

Common strategies have been proposed to protect privacy such as de-identification (Comess et al., 2020; Aebi et al., 2021), anonymization, (Comess et al., 2020; Gilbert et al., 2020; Aebi et al., 2021) and geo-masking (Comess et al., 2020; Aebi et al., 2021). However, these strategies face several limitations such as the complex language of privacy policies (Aiello et al., 2020), the lack of transparency about the protection mechanism used (Casanovas et al., 2017), the overall cost of the protection mechanisms (Zhang et al., 2017; Kayaalp, 2018), the use of protection mechanism more adequate for “small data” rather than BD (Wang and Alexander, 2020; Sun et al., 2020), and the ambiguous status of sensible data shared on social media (Celedonia et al., 2021; Gilbert et al., 2020).

The value of privacy conflicts with the possible benefits associated with using BD and AI in health-related contexts (Sparrow and Hatherley, 2019; Cool, 2016; Goodman, 2020; Salerno et al., 2017; Gilbert et al., 2019; van Heerden et al., 2020; Yeung, 2018; Igual et al., 2013). During the COVID pandemics, empirical data have shown that, for certain people, the loss of privacy was perceived as a trade-off for public health (Liu and Graham, 2021; Degeling et al., 2020). Aside from greater public health outcomes and prevention (Dolley, 2018; Alemayehu and Berger, 2016; Aiello et al., 2020; Horvitz and Mulligan, 2015; Roberts, 2019; Raza and Luheshi, 2016; Adkins, 2017; Mootz et al., 2020; Hodgson et al., 2020; Mahlmann et al., 2017), authors suggest that the promotion of scientific innovation could outweigh privacy (Heitmueller et al., 2014; Balas et al., 2015; Manrique de Lara and Pelaez-Ballestas, 2020; Salerno et al., 2017; Liu and Bressler, 2020; Comess et al., 2020; Terry, 2014; Wyllie and Davies, 2015).

#### 3.3.2 Consent

The use of BD and AI in health-related contexts raises issues of free and informed consent (Stylianou and Talias, 2017; Wang and Alexander, 2020; Galetsi et al., 2019; Sparrow and Hatherley, 2019; Vollmer Dahlke and Ory, 2020; Ahmed et al., 2020; Canaway et al., 2019; Zhang et al., 2017; Manrique de Lara and Pelaez-Ballestas, 2020; Ossorio, 2014; Samuel and Derrick, 2020; Xafis et al., 2019; Casanovas et al., 2017; Gossec et al., 2020; Pepin et al., 2020; Deshpande et al., 2019). Using the populations’ data without their consent could weaken trust in institutions and researchers (Tsai and Junod, 2018). Conversely, transparent consent practices could foster trust, especially in underrepresented groups (Zou and Schiebinger, 2021). Paradoxically, there may be too few or too many moments for consent in BD and AI technologies (van Deursen and Mossberger, 2018; Montgomery et al., 2018). Also, consent regulations vary between countries and cultures (Sanchez M and Sarria-Santamera, 2019). Consent is linked to issues of accessibility, as it can enable individuals to control the use of their data (Andanda, 2019). However, informed consent does not necessarily grant people control over their data (Ienca et al., 2018). Thus, the question of control over one’s data may be more important than questions regarding consent (Kostkova et al., 2016).

Several situations compromising consent have been identified in the literature. Consent issues may arise when data is used for purposes that have not been consented to by individuals (Bellazzi, 2014) because the intervention is aiming at large populations (Thorpe and Gray, 2015a; Sanchez M and Sarria-Santamera, 2019; Ienca et al., 2018; Gilbert et al., 2020), such as public health surveillance (Aiello et al., 2020; Conway, 2014; Samuel and Derrick, 2020; Genevieve et al., 2019; Gilbert et al., 2019; Thorpe and Gray, 2015b; Park, 2021), the creation of integrated databases (Wyllie and Davies, 2015), electronic healthcare predictive analysis (Mootz et al., 2020), the linkage of data (Vogel et al., 2019; Bates et al., 2018; Salerno et al., 2017; Joda et al., 2018), biobanking (Docherty and Lone, 2015; Sanchez M and Sarria-Santamera, 2019; Cool, 2016; Mootz et al., 2020; Tigard, 2019; Snell, 2019; O’Doherty et al., 2016; Shah and Khan, 2020; Wyllie and Davies, 2015), and public health emergencies (Shachar et al., 2020). Another difficulty may come to consent for data already publicly available (Rosen et al., 2020). Passive data collection with sensors in the environment or assistive technologies (Kernaghan, 2014; Bennett, 2019; Grigorovich and Kontos, 2020; Miller, 2020; Ienca et al., 2016; Ienca et al., 2018) may also prevent consent mechanism (Manzeschke et al., 2016; van Heerden et al., 2020) and make individual unaware that personal data are collected. Registries, health data record and electronic health records raise the issue of the difficulty to opt-out of these platforms or to be aware of their secondary use (Balas et al., 2015; Joda et al., 2018; Tsai and Junod, 2018; Nakada et al., 2020) by third parties (Kerr et al., 2018; O’Doherty et al., 2016). This situation is complicated if data have already been anonymized (Joda et al., 2018).

Social networks are also sensible platforms for obtaining authentic informed consent. Personal data on these platforms can be of great interest for different actors such as HCPs (Terrasse et al., 2019), healthcare systems (Young, 2018), data brokers (Horvitz and Mulligan, 2015) and researchers (Althobaiti, 2021; Conway, 2014). In principle, public domains are open to data mining (e.g., public health research), but what constitutes a public domain is less clear regarding social media (Vayena et al., 2015; Young, 2018; Wyllie and Davies, 2015). Consent processes on these platforms can be difficult to understand (Aiello et al., 2020; Nebeker et al., 2019; Villongco and Khan, 2020; Gilbert et al., 2020) and people may be nudged to consent mechanically (Terrasse et al., 2019; Mikal et al., 2016).

For some, respecting individual rights implies consent mechanisms (Vayena et al., 2015), but the inability to use data from some populations limits its utility (Cahan et al., 2019). This raises the more general question as to whether individual consent should be sought before using BD and AIS given their potential benefits (Gilbert et al., 2019) or if we should incentivize for the voluntary donations of sensitive data (Tigard, 2019). Some authors argue that, at least, some data should be available without individuals’ consent because of its utility for efficient public health interventions (Balas et al., 2015; Gilbert et al., 2019). Thus, it may be justified to do public health surveillance without consent (Aiello et al., 2020). Also, “[i]nsistence on formal consent for big data research could cause wider societal harm, as the participation bias which might arise could skew the data to such an extent as to make results inaccurate or meaningless” (Docherty and Lone, 2015). In fact, patients may not be aware of the potential of their medical data for research and of the barriers to access it (Machluf et al., 2017) or they may consent only if they feel it is in their interest (Yang and Chen, 2018). Broadly, some laws may allow the divulgation of health information for public health activities without requiring individual consent (Thorpe and Gray, 2015b).

To respond to these issues raised by AIS and BD, new forms of consent are needed (Andanda, 2019; Zhang et al., 2017; Genevieve et al., 2019; Tigard, 2019). Broad consent is an option explored (Hemingway et al., 2018), but its universal applicability is questioned (Howe and Elenberg, 2020; van Heerden et al., 2020). Other options include meta-consent (Sanchez M and Sarria-Santamera, 2019), opt-out and dynamic consent (Andanda, 2019), a trust-based approach to consent (Pickering, 2021), and e-consent (Genevieve et al., 2019). The latter has many drawbacks: users may not read or understand the information provided in the e-consent form; there is no interaction between them and the researcher; and it is difficult to ascertain the individual’s identity (Genevieve et al., 2019). Another type of consent, opt-in consent, may promote informed consent but may result in selection bias, particularly with vulnerable populations (Bates et al., 2018; Evans et al., 2020).

#### 3.3.3 Responsibility, accountability, and liability

AIS raises several issues at the intersection responsibility, accountability and liability (Samuel and Derrick, 2020). Authors ask who is responsible (Ladner and Ben Abdelaziz, 2018), and who is responsible for ensuring the reliability of AIS and their data (Goodman, 2020)? Accountability is connected with “quality, standards, and ethics” (Goodman, 2020) and can conflict with other public health values such as the maximization of benefits (Rosen et al., 2020). In the literature, the term “responsibility” can be used interchangeably with “accountability” and “liability.” In the most general sense, “responsibility” means to hold someone responsible for an act (Cornock, 2011). For its part, “accountability” “simply means to be called to account” (Cornock, 2011). Liability can be seen as a legal accountability which implies to the obligation of giving an account the possibility of sanction (Cornock, 2011). Although different concepts, it is not clear if such distinctions are maintained in the literature.

For the authors, it is clear that AIS in healthcare blur the notion of professional responsibility (Manrique de Lara and Pelaez-Ballestas, 2020). Who should be held accountable and who should be responsible in case an intervention based on AIS harms individuals (Sparrow and Hatherley, 2019; Jones et al., 2020; Manrique de Lara and Pelaez-Ballestas, 2020)? This problem of responsibility comes from the capacity for AI to have an agency or not (Sparrow and Hatherley, 2019). The main tendency is to make HCPs “in charge” when using medical AIS (Manrique de Lara and Pelaez-Ballestas, 2020). Because there is always a human in the loop, humans are responsible for adverse consequences (Lanier et al., 2020; Lupton and Jutel, 2015). In case of an adverse consequence resulting from the use of an AIS, we can always assess whether the HCP’s choice to use this technology was reasonable (Sparrow and Hatherley, 2019) and AIS should be held to the same degree of accountability and effectiveness as other medications and devices (Tang et al., 2018).

Outside the narrow medical field, the literature points toward several example of unclear responsibility (Carney and Kong, 2017). For example, carebots interacting with people with dementia implies agents that are not fully competent (Ienca et al., 2016). Social networks have their share of ambiguity. They can offer health related services but are not considered responsible HCP (Celedonia et al., 2021); they offer data for researchers, but they are not responsible for protecting users privacy (Andanda, 2019). Also, it is not clear who should be held liable for a device malfunction and adverse consequences (Kerr et al., 2018; Sparrow and Hatherley, 2019), or for data lack of quality and security (Kern et al., 2016; Casanovas et al., 2017): the HCP, researchers (Samuel and Derrick, 2020; Ballantyne, 2019), the developers (van Deursen and Mossberger, 2018), the manufacturer, corporations owning the technology (Andanda, 2019), the designer, purchaser of the AI, shareholders, or the AI itself (Sparrow and Hatherley, 2019)? This led Mahlmann and collaborators (Mahlmann et al., 2017) to argue that accountability needs to be at multiple levels because data used in health come from different fields with different legal responsibilities with different forms of access.”

#### 3.3.4 Transparency

Making AIS (and the reasons for their use) transparent is a central issue in the literature (Lee and Yoon, 2017; Althobaiti, 2021; Horvitz and Mulligan, 2015; Li and Cong, 2021; Godfrey et al., 2020; Machluf et al., 2017; Tupasela et al., 2020; Vayena et al., 2015; Kirtley and O’Connor, 2020). Transparency is an important value for both AIS and population health (Kim et al., 2017; Rosen et al., 2020) as it is an essential mechanism to guarantee accountability, public support, inclusion, and trust (Sanchez M and Sarria-Santamera, 2019; Cordeiro, 2021; Ballantyne, 2019; Kostkova et al., 2016; Vayena et al., 2015). Transparency implies “openness to public scrutiny of decision-making, processes, and actions.” (Xafis et al., 2019) Transparency issues are critical at two different levels.

First, the opacity of BD-based technologies can make it impossible for external actors to understand the value of the information (Roberts, 2019). This uncertainty regarding data may occur at each step of data processing: from data collection (Morgenstern et al., 2021; Murphy et al., 2021; Evans et al., 2020; van Deursen and Mossberger, 2018; Manzeschke et al., 2016; Liu and Graham, 2021; Ajunwa et al., 2016), its storage (Manzeschke et al., 2016; Ajunwa et al., 2016), its ownership (Kostkova et al., 2016; Ajunwa et al., 2016), its sharing (Murphy et al., 2021; Manzeschke et al., 2016; Cool, 2016; Deshpande et al., 2019) to its uses (Canaway et al., 2019; Li and Cong, 2021; Evans et al., 2020; van Deursen and Mossberger, 2018). Data transparency is important for health organizations (Leyens et al., 2017) as well for patients (Ahmed et al., 2020; Ienca et al., 2016) and is seen as responsible data management (Cordeiro, 2021). However, data transparency must be balanced with other values such as confidentiality (Straw, 2021; Raza and Luheshi, 2016), privacy (Kostkova et al., 2016; Mohr et al., 2017), and innovation (Horgan and Ricciardi, 2017; Babyar, 2019).

Second, a common aspect of the transparency issue is AI’s black box problem; in other words, the fact that its results are not explainable (Ahmed et al., 2020; Morgenstern et al., 2021; Kee and Taylor-Robinson, 2020; Terrasse et al., 2019; Liu and Bressler, 2020; Murphy et al., 2021; Thomasian et al., 2021; Couch et al., 2020; Montgomery et al., 2018; Kasperbauer, 2021; Lanier et al., 2020; Pepin et al., 2020; Wongkoblap et al., 2017; Delpierre and Kelly-Irving, 2018). In the clinical context, this may impair an HCP’s capacity to identify and mitigate risks for patients, and to discuss and interpret the results (Luk et al., 2021; Sparrow and Hatherley, 2019). More generally, the black box problem may cause a loss of control for data scientists and the population (Delpierre and Kelly-Irving, 2018). When data is not made transparent, algorithmic outcomes cannot be reproduced and checked for accuracy (Tan et al., 2020). Many authors argue that AIS should be more transparent and explainable (Ahmed et al., 2020; Lodders and Paterson, 2020; Fulmer, 2019; Benke and Benke, 2018) and that developers should be transparent about the evidence supporting their product (Kirtley and O’Connor, 2020; Ienca et al., 2018), their underlying assumptions (Delpierre and Kelly-Irving, 2018), theirs ends (Delpierre and Kelly-Irving, 2018), the product’s risks, and its benefits (Kirtley and O’Connor, 2020). However, others argue that making all AIS transparent could be unrealistic because of its complexity and its understandability by only few experts (Terrasse et al., 2019). Yet others emphasize that the health sector is already full of “black boxes” (Sparrow and Hatherley, 2019) leading to the question if we may be able, 1 day, to trust black box healthcare (Manrique de Lara and Pelaez-Ballestas, 2020).

#### 3.3.5 Trust

A lack of transparency can lead to trust issues (Sparrow and Hatherley, 2019; Straw, 2021) at different levels. At the clinical level, the incapacity to explain the results of an AIS may lead an HCP to lose trust in the system (Chen and See, 2020). The deterioration of the patient-HCP relationship can reduce the quality of healthcare services (Cordeiro, 2021) and deter patients from disclosing certain information and participating in research (Manrique de Lara and Pelaez-Ballestas, 2020; Shah and Khan, 2020; Nageshwaran et al., 2021; Rehman et al., 2022). Furthermore, trust helps clinicians and patients approve of the conclusion of an AIHT (Sparrow and Hatherley, 2019; de Graaf et al., 2015; Noorbakhsh-Sabet et al., 2019). Conversely, automatic decision-making processes could be perceived as trustworthy because of their accuracy and impartiality (Araujo et al., 2020).

At the population level, trust is a relational notion bonding citizens and institutions (Ballantyne, 2019). It facilitates the social acceptability of technologies or health practices (Bellazzi, 2014; Sanchez M and Sarria-Santamera, 2019; Balas et al., 2015), the engagement and involvement of communities in AIS development (Hunt et al., 2020; Dankwa-Mullan et al., 2018) and the cooperation of citizens in health initiatives (Ballantyne, 2019; Naudé, 2020), such as public health surveillance systems (Gilbert et al., 2019; Lodders and Paterson, 2020), and biobanking (Colloc, 2015). Trust may also be necessary to address discrimination concerns related to technologies using personal and genetic data (Trein and Wagner, 2021).

For these reasons, trustworthiness is an important ethical value for the implementation of these technologies (Althobaiti, 2021; Rosen et al., 2020; Samuel and Derrick, 2020; Samuel and Derrick, 2020; Xing et al., 2021; Prosperi et al., 2018; Mahlmann et al., 2017). More specifically, patients and the public must trust that their data is used according to their wishes (Andanda, 2019; Lodders and Paterson, 2020), that their privacy is respected (Balas et al., 2015; Abdulkareem and Petersen, 2021; Ienca et al., 2018; Thorpe and Gray, 2015b; Mohr et al., 2017) that data is safe (Fornasier, 2019; Salerno et al., 2017; Shahid et al., 2021; Tsai and Junod, 2018; Wyllie and Davies, 2015; Ienca et al., 2018) and that there are regulations governing the use of data (Tan et al., 2020). However, building and maintaining public trust is challenging (Aiello et al., 2020; Conrad et al., 2020; Hemingway et al., 2018), especially for minority groups (Zhang et al., 2017). Trust can be weakened when organizations sell data to third parties (pharmaceutical, insurance, etc.) for financial gain (Canaway et al., 2019; Gilbert et al., 2019; Kostkova et al., 2016; Tupasela et al., 2020). Weak oversight of such data-sharing (Sanchez and Sarria-Santamera, 2019; Villongco and Khan, 2020), lack of data accuracy, biases or misleading conclusions (Aiello et al., 2020; Grigorovich and Kontos, 2020; Dolley, 2018; Goodman, 2020; Vayena et al., 2015; Gilbert et al., 2019; Igual et al., 2013) and bad communication strategies (Nebeker et al., 2019) can also lead to a crisis of confidence in the technologies (Heitmueller et al., 2014). Rebuilding trust after a loss from the public can be challenging (Bates et al., 2018).

#### 3.3.6 Social acceptability

As discussed above, trust facilitates social acceptability, which is a “primary concern” related to using AIS and BD (Tang et al., 2018). This notion is associated with popular support, which is necessary for data collection (Katapally, 2020), the successful implementation of AIBD technologies (Mootz et al., 2020; Prosperi et al., 2018; Esmaeilzadeh, 2020; Salas-Vega et al., 2015) and the viability of product development or research endeavors (Canaway et al., 2019; Cool, 2016). Little research has explored users’ acceptability of AIS and BD technologies (Wongkoblap et al., 2017; Igual et al., 2013), but some articles have shown that public attitudes toward these technologies may vary depending of their aim (Nakada et al., 2020), data ownership (Ienca et al., 2018) and the perception of subpopulations (Heitmueller et al., 2014). Furthermore, people might be more willing to tolerate data sharing and privacy breaches if they consider that it is for the common good (Gilbert et al., 2020) and if they understand what AIS can offer them personally in terms of health outcomes (Kelly et al., 2020). On the HCP’s side, various factors can influence their support for AIS such as the characteristics of the technology, their knowledge, their opinions, external factors (e.g., patient and health professional interaction), and the organizational capacity to implement it (Kelly et al., 2020). During the COVID-19 pandemic, the fear of infection and death affecting individuals and their families has led to a growing understanding of the importance of public health and therefore contributed to increasing the acceptability of health surveillance (Couch et al., 2020). The pandemic also contributed to an acquired familiarity with telemedicine services and digital health platforms (Ho et al., 2020). However, if AIS do not meet ethical standards, stakeholders might be opposed to their implementation and therefore those technologies will not reach the populations for which they were designed (Abramoff et al., 2021).

## 4 Discussion

This review synthesized the state of knowledge on the ethical issues of the combined use of AIS and BD in the context of population health. The literature suggests that these technologies may affect every component of population health. At this stage, the literature still debates if the technologies will lead to positive or negative outcomes. Positive outcomes are mostly conceived as an optimization of existent health and research activities. Those who focus on negative outcomes are concerned about communities potentially becoming overly reliant on digital systems as a result of the anticipated AI revolution. An important challenge will be distribution of the benefits and burdens of these technological transformations. There are strong voices anticipating that this distribution will be unfair between populations and inside populations and that it will reinforce prevailing inequities.

This synthesis reveals the need for a balanced perspective, as the potential benefits of AIS and BD, such as precision public health and improved decision-making, are accompanied by substantial ethical risks. A more nuanced approach to interpreting results is essential, particularly one that explicitly addresses both benefits and risks with real-world examples. For instance, initiatives like the “AI for Good” projects by global organizations highlight pathways for leveraging AI ethically, particularly in underrepresented communities.

Aside from these outcomes, we can expect that AIS and BD will affect upstream determinants of health. Because of the ubiquitous nature of BD and AI (Benke and Benke, 2018), these technologies may penetrate every aspect of our existence and, by extension, every element contributing to the overall health of communities. Regardless of this baffling projection, our review encourages to look at specific patterns of health determinant that are considered, to this day, more sensitive to the influence of AI and BD technologies. However, these upstream effects also raise critical concerns about data access and ownership, particularly in the context of global inequities. For example, data collected in LMICs often benefits high-income settings disproportionately, perpetuating patterns of digital colonialism. Interventions addressing these disparities might include creating localized data governance frameworks that empower LMIC stakeholders to oversee and benefit from the use of their data. Developing equitable access to AI training and infrastructure is another pathway to mitigate these issues.

If we look in more detail to the effect of AIS and BD on the determinants of health, the first pattern of health determinants our review identified relates to healthy behaviors. Authors are dubious if the technologies will assist individuals in adopting health behaviors personalized to their conditions. To attain this goal, developing digital and ethical literacy in all segments of the population appears to be an inevitable avenue. A similar doubt persists in the discussion on AIS and BD effects regarding the access and quality of healthcare, the second pattern of health determinants identified in the review. On the one side, the literature argue that the technologies will assist HCPs in their daily tasks, while on the other side, they will accentuate the workload of HCPs and contribute to their deskilling because of their increased dependency on the technology. Further, the impact on health behaviors highlights the importance of patient trust and engagement. Enhancing transparency in AIS can improve trust and empower patients. For example, using explainable AI (XAI) systems in clinical decision-making could foster a stronger relationship between healthcare professionals (HCPs) and patients, as it allows for clearer communication of how decisions are reached. Implementing dynamic consent models could also enhance patients’ control over their data, addressing trust and autonomy concerns simultaneously.

The third pattern deals with the idea that, with the growing recourse to digital health apparatuses, data infrastructures will become a new determinant of population health. Who control data and has access to it will shape profoundly how the benefits and burdens of the technologies will be distributed globally. To ensure equitable outcomes, international data-sharing agreements must incorporate ethical safeguards. For instance, mechanisms for broad but controlled access to non-proprietary datasets, akin to the open science movement, could promote collaboration while protecting sensitive information. Moreover, innovative models like “data trusts,” where communities collectively manage their data, could provide an ethical way to balance privacy, transparency, and accessibility.

The last component of population health relates to interventions and policies. From an ethical perspective, population health interventions are essentially examined on their capacity to generate a complex trade-off between health goals, economic profit, scientific innovation, and collective moral values. The literature advise that we should give a particular attention to how any intervention or policy value privacy protection, free and informed consent, responsibility, and transparency. Respecting these values will contribute to two other inextricable values that are trust and social acceptability, which are essential in the implementation of all population health interventions and policies. Transparency is particularly critical in overcoming the “black box” issue prevalent in many AIS. Embedding requirements for explainability in AI regulatory frameworks could improve not only clinical decision-making but also public trust. Policymakers should look to best practices from other domains, such as the EU’s General Data Protection Regulation (GDPR), which could inspire guidelines on managing data and ensuring accountability.

An additional domain warranting attention involves the epistemological assumptions underpinning AI and BD systems and the statistical fragilities embedded in data-driven models. Much of the literature we reviewed does not critically engage with the capacity of BD and AI to produce valid insights through sheer volume, pattern recognition, and algorithmic refinement. Yet, epistemologically, these systems often prioritize correlation over causation, prediction over explanation, and model fit over interpretive depth; raising foundational questions about what kind of knowledge they generate and how it should inform population health decisions (Leonelli, 2019). Furthermore, the statistical reliability of these systems is subject to multiple threats, including overfitting, selection bias, spurious correlations, and algorithmic opacity (Stiglic et al., 2020), which can lead to “hallucinations,” especially with large language models, which can have extremely significant impacts in high-stake setting such as medicine (Bélisle-Pipon, 2024). In population health, where interventions rest on population-level inferences, such errors may propagate systemic misclassifications or misleading policy signals. A theory-driven approach, integrating causal inference, domain expertise, and interpretive reasoning, remains critical to counterbalance the limits of purely data-driven methods (Cavique, 2024; Pearl and Mackenzie, 2018). The absence of this epistemic reflection risks reinforcing technocratic approaches that obscure value-laden judgments beneath a veneer of objectivity. Future ethical appraisals must scrutinize not only what AI and BD do, but also how they know.

Overall, the literature speculates that AIS using BD will affect population health in an unprecedent manner and with ethical consequences. There are no components of population health that will be immune to the penetration of these technologies in the numerous activities of the actors in the field. It is anticipated that the technologies will shape the determinants of health as well as the interventions and policies aimed at working positively on these determinants.

### 4.1 Engaging with actionable insights

To move beyond theoretical considerations, actionable recommendations may support stakeholder engagement in answering these questions. Policymakers, developers, healthcare professionals, and researchers each have a role in ensuring the ethical deployment of AIS and BD. Table 4 outlines specific actions for these groups, aligned with key ethical principles and lifecycle phases (Collins et al., 2024).

**Table 4:** Actionable insights for ethical governance of AIS and BD.

Stakeholder group	Actionable insight	Lifecycle phase addressed
Policymakers	Establish regulations for explainable AI (XAI) to ensure transparency and accountability.	Purpose, development
	Incentivize the creation of “data trusts” to empower communities to manage their data collectively.	Data, development
	Mandate periodic audits of AIS for bias and inequity during deployment and operation.	Validation
Developers	Incorporate diverse, representative datasets to minimize algorithmic bias.	Data, development, generalization
	Design AI systems with user-friendly interfaces to enhance digital literacy and usability.	Development, application
	Plan for decommissioning by ensuring data and algorithms are securely retired or repurposed ethically.	Decommissioning
Healthcare professionals	Train HCPs on the use and limitations of AIS to foster informed, balanced decision-making.	Application
	Advocate for shared decision-making models that integrate AIS insights with clinical expertise.	Application
Researchers	Use participatory research methods to include marginalized populations in AI and BD studies.	Purpose, data
	Develop metrics to evaluate the social acceptability and trustworthiness of AIS interventions.	Application, validation
Patients	Promote digital literacy programs to help patients understand and engage with AIS in healthcare.	Application, generalization
	Develop patient-centered feedback mechanisms for AIS to ensure systems align with patient values.	Application, development
	Advocate for inclusion in co-design processes to align AIS with real-world patient needs.	Purpose, development
General public	Organize public consultations to gather community perspectives on ethical concerns in AIS deployment.	Purpose, development, application
	Create educational campaigns to increase awareness of data privacy, consent, and ethical AI practices.	Monitoring, feedback
	Provide accessible mechanisms for individuals to inquire about or opt out of data use in AIS systems.	Feedback, application

Table 4 seeks to supplement the review findings with a structured summary of ethical governance strategies, organized by stakeholder group, type of intervention, and the specific lifecycle phase of AIS and BD systems. This kind of lifecycle mapping has been increasingly recommended to operationalize ethical principles across the development, implementation, and decommissioning of AI technologies (Floridi et al., 2018; Collins et al., 2024). The table foregrounds concrete roles (from data stewardship and explainability enforcement to bias audits and participatory co-design) offering a modular governance framework adapted to both institutional and technical contexts (Pacia et al., 2024; Morley et al., 2020). Developers, clinicians, patients, policymakers, and civil society actors are presented not as passive recipients of ethical guidance, but as active agents responsible for aligning technological deployment with public values (Vayena et al., 2018; Bélisle-Pipon and Victor, 2024). Crucially, we emphasize that governance must extend beyond static principle-based declarations, incorporating iterative accountability mechanisms throughout the system’s operational life (Mittelstadt, 2019). Table 4 is designed as both a synthesis and a practical entry point for translating ethics into targeted interventions at specific moments in the AI and BD lifecycle.

The review results resonate with other reviews on the ethics of AI and BD in healthcare (Morley et al., 1982; Murphy et al., 2021; Bélisle-Pipon et al., 2021; d’Elia et al., 2022). However, our results take their distance from a perspective centered on individual, medical and clinical care, to adopt the more global perspective of population health and upstream determinants of health. There are no clearcut demarcations between individual and population health, but technologies such as AI and BD generate their own blurring of these distinctions by offering the technological means to move from set of data pertaining to large group of individuals to conclusion applying to a specific individual. This blurring, or what Shipton and Vitale refer to a “politic of avoidance” (Shipton and Vitale, 2024), should not obscure that the technologies may affect entire populations and health determinants in a subtle manner as suggested by the present review.

### 4.2 Limits

While this review provides a comprehensive synthesis of the ethical issues surrounding AI and big data in population health, it is important to acknowledge certain limitations that could impact the breadth and applicability of the findings. One of the most significant limitations is the temporal scope of the literature considered. The review synthesizes articles published up to November 2021, meaning that it does not account for advancements, challenges, or ethical insights that have emerged in the last 4 years—a period characterized by rapid technological evolution and significant global events.

The exclusion of literature beyond 2021 omits critical developments in the field, such as the rise of generative AI systems, including large language models like GPT (e.g., ChatGPT’s GPT-4), which have revolutionized AI applications across industries, including healthcare. These systems have introduced new ethical dimensions, such as the propagation of misinformation, explainability issues, and risks of misuse in clinical and public health contexts. These topics, largely absent from the pre-2021 literature, represent key areas of concern that would likely require attention in an updated analysis. Additionally, the review does not address the broader implications of post-pandemic technological advancements. The COVID-19 pandemic significantly accelerated the adoption of AI technologies for public health surveillance, vaccine distribution, remote patient monitoring, and digital contact tracing. The normalization of such technologies has raised new ethical questions around privacy, consent, and equity, particularly in how these tools have been used to monitor populations at scale. These shifts are likely underexplored in the reviewed literature due to the timing of the search.

Since 2021, there have also been important regulatory and ethical developments, such as the European Union’s Artificial Intelligence Act and a growing emphasis on data sovereignty globally. These developments reflect a shift toward formalized governance frameworks that seek to address many of the concerns raised in this review. However, the analysis in this study predates these frameworks, which limits its ability to reflect the current regulatory landscape and its implications for population health. Equity and inclusion have also emerged as prominent themes in recent AI research. Advances in methodologies for debiasing algorithms, participatory AI design, and equity audits have provided tools to promote fairness and inclusivity in AI systems. These tools, while critical to addressing disparities in healthcare, are underrepresented in the body of literature included in this review. Similarly, the environmental impact of AI, particularly the carbon footprint of training large-scale models, has become an increasingly important ethical consideration that was likely not a major focus of studies published before 2022.

This temporal limitation risks presenting an incomplete or outdated understanding of the ethical landscape of AI and big data in population health. Omitting key developments from recent years could lead to an overemphasis on challenges identified in earlier stages of technological maturity while neglecting the ethical issues arising from newer applications and regulatory responses. It also limits the capacity to provide actionable insights for addressing contemporary ethical dilemmas in the field. To address this limitation, future research must prioritize updating the review to include studies published since 2021. Incorporating more recent developments will ensure that the findings remain relevant and responsive to current trends. Additionally, establishing a mechanism for periodic review updates, such as every two to 3 years, could help maintain the relevance of the synthesis over time. Engaging with practitioners and experts working on the front lines of AI ethics in healthcare could further complement the literature, adding real-world insights into the ongoing evolution of these technologies.

### 4.3 Future research

Considering the limitations of our review process, we would like to conclude by pointing avenues of research on the ethics of AIS and BD in population health that have been discussed since the end of our data analysis (Couture and Bélisle-Pipon, 2023).

Future research will have to integrate the effect of AIS and BD on other important health determinants. For example, policymakers will have to recognize the environmental cost of AIS and BD infrastructures and their consequences on the health of communities (Couture et al., 2023). The disinformation capacity of AI represents another serious threat for the implementation of any health interventions, but also for the stability political institutions (Federspiel et al., 2023). The use of AIS in warfare will also have to be considered as well as the health outcomes of the global transformation of employment and workplace conditions that are taking place with the diffusion of AIS (Federspiel et al., 2023).

To complete this task, AI ethics will need to widen its scope and follow the lead of population health in evaluating the deployment of AI and BD. Future research will need to answer three essential ethical questions: Do the interventions and policies using these technologies have a positive effect on patterns of health determinants? Do this positive outcome is obtained while sufficiently respecting collective moral values? Do the amalgamation of all these specific interventions and policies contribute, at the end, to a just society?

In answering these questions, a deeper integration of cross-disciplinary frameworks is essential. For example, justice-oriented approaches from bioethics could be combined with data science methodologies to develop predictive models that prioritize fairness and equity. Stakeholder engagement, especially involving marginalized populations, should become a cornerstone of both research and implementation to ensure that technologies align with societal values.

## Data Availability

The data analyzed in this study is subject to the following licenses/restrictions: The dataset is mostly qualitative. Please contact the corresponding author. Requests to access these datasets should be directed to vincent.couture@umontreal.ca.
