# Supplementary material for: Ethical issues raised by artificial intelligence and big data in population health: a scoping review
Source: Front Sociol. 2025 Sep 9;10:1536389. doi: 10.3389/fsoc.2025.1536389 (PMC12454051; doi:10.3389/fsoc.2025.1536389)
Supplement: Supplementary file 1 [file Data_Sheet_1.pdf]

Supplementary Material

Charting the Data

Our research yielded articles published between 2002 and 2021. The number of publications increased greatly in the recent years, with twice as many articles published in 2020 (n=72) as in 2019 (n=36) (see Figure 2). Because our literature review was updated on November 24, 2021, it underestimates the number of articles published in 2021. However, we would have expected more articles being published in 2021 because of the COVID-19 pandemic and the development of multiple BD and AI surveillance tools.

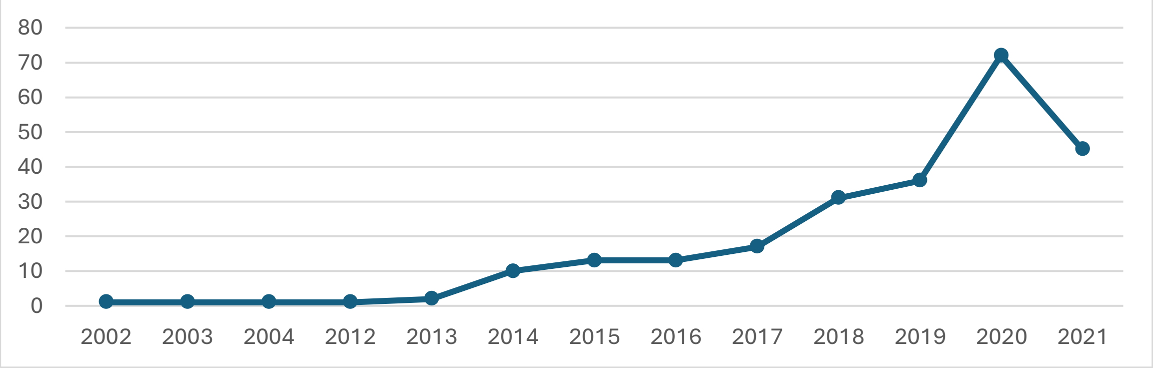

Figure 2: Number of articles published by year

Figure 3 shows the regions (as per the United Nations geographic regions) associated with papers' first author's first affiliation. N=115 papers were published by first authors based in North America (United States: n=95; Canada: n=20). The second most represented region is Europe (n=75), with the UK accounting for a third of European publications (n=25) Western countries (North America and Europe) account for 78% of publications.

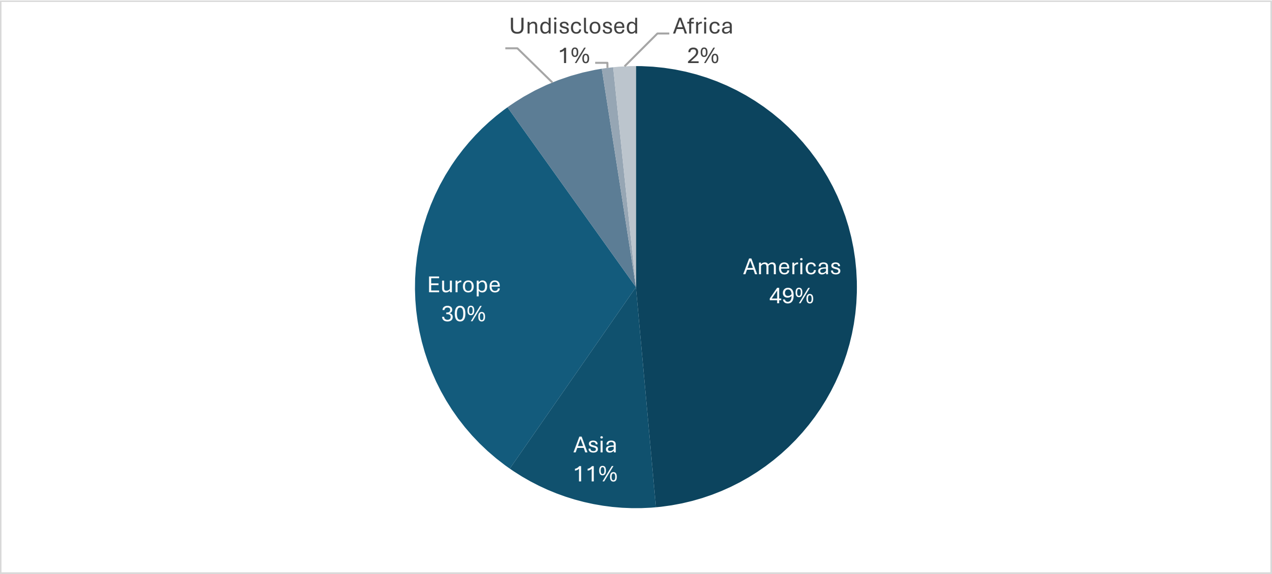

Figure 3: Percentage of publications by region

The majority of articles pertained to the health sciences (n=114; 47%), followed by ethics, law and policy (n=87; 36%), social sciences and humanities (n=25; 10%) and computer sciences (n=18; 7%) (what we termed "publication domain"; see figure 4). Thus, the ethical issues of AIS and BD in population health concern various academic disciplines albeit to different degrees.

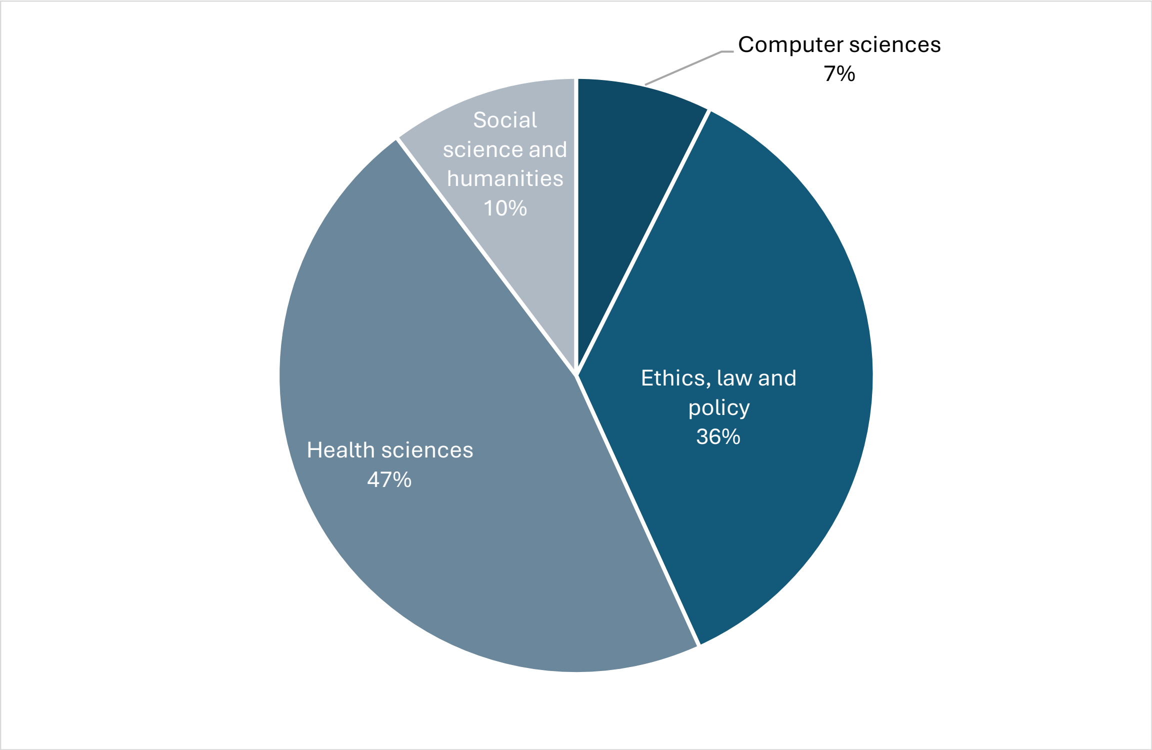

Figure 4: Distribution of the publication domain

The most common application of the technologies found in the articles reviewed was public health (n=99) followed by clinical applications (n=86), reflecting our inclusive definition of population health (see Figure 5). However, many articles referred to more than one application, which is not reflected in the table above.

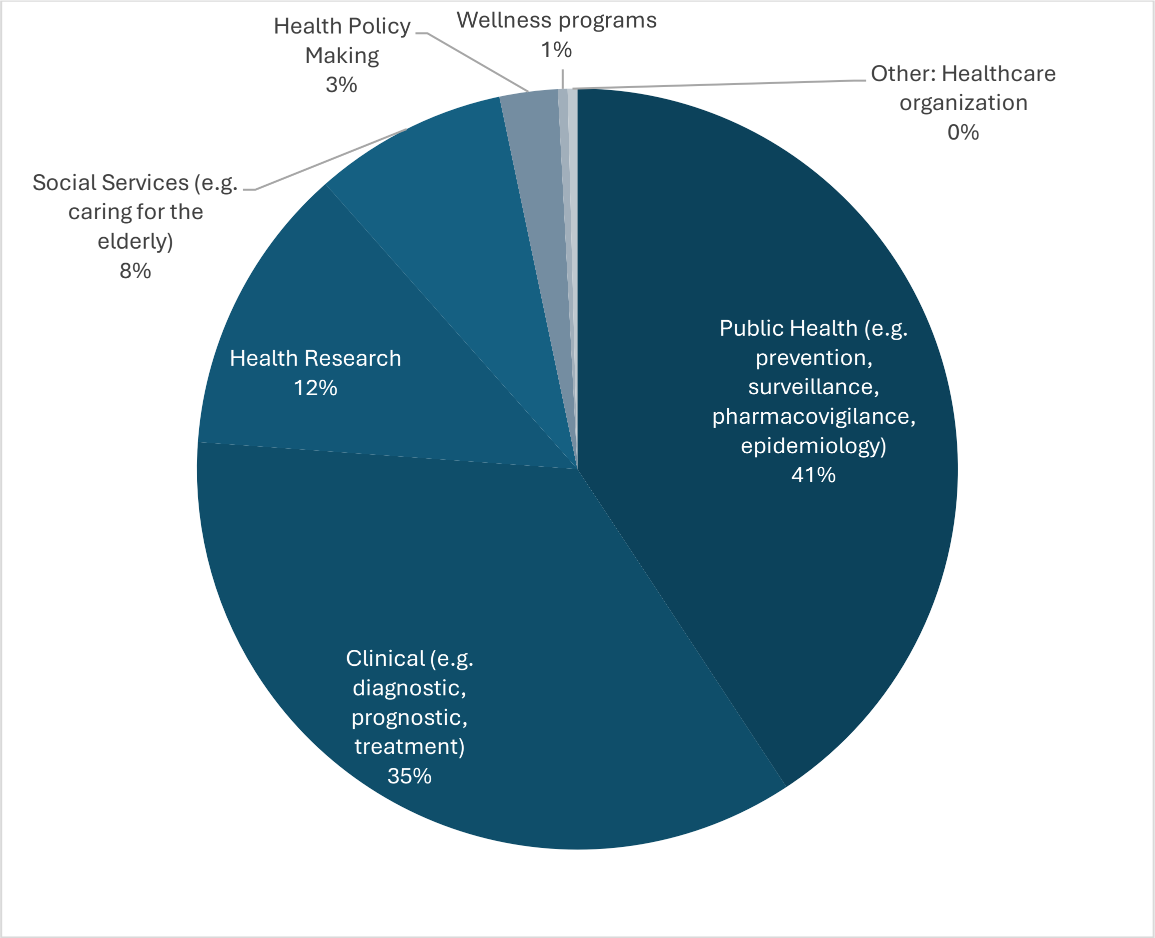

Figure 5: Distribution of the BD and AI applications in population health
